# Laser-Induced Metal–Organic Framework-Derived Flexible Electrodes for Electrochemical Sensing

**DOI:** 10.1021/acsami.4c18243

**Published:** 2025-01-06

**Authors:** Beatrice De Chiara, Fulvia Del Duca, Mian Zahid Hussain, Tim Kratky, Pritam Banerjee, Sarah V. Dummert, Ali Khoshouei, Nicolas Chanut, Hu Peng, George Al Boustani, Lukas Hiendlmeier, Joerg Jinschek, Rob Ameloot, Hendrik Dietz, Bernhard Wolfrum

**Affiliations:** † Neuroelectronics, Munich Institute of Biomedical Engineering, Department of Electrical Engineering, School of Computation, Information and Technology, Technical University of Munich, Hans-Piloty-Str. 1, 85748 Garching, Germany; ‡ Chair of Inorganic and Metal−Organic Chemistry, Department of Chemistry, School of Natural Sciences, Technical University of Munich, Lichtenbergstr. 4, 85748 Garching, Germany; § Physical Chemistry with Focus on Catalysis, Department of Chemistry, School of Natural Sciences, Technical University of Munich, Lichtenbergstr 4, 85748 Garching, Germany; ∥ National Centre for Nano Fabrication and Characterization (DTU Nanolab), Technical University of Denmark, Fysikvej 307, DK-2800 Kongens Lyngby, Denmark; ⊥ Laboratory for Biomolecular Nanotechnology, Department of Biosciences, School of Natural Sciences, Technical University of Munich, Am Coulombwall 4a, 85748 Garching, Germany; # Center for Membrane Separations, Adsorption, Catalysis, and Spectroscopy (cMACS), KU Leuven, 3001 Leuven, Belgium

**Keywords:** laser-induced graphitic carbon, UV laser, metal−organic frameworks, MOF derivatives, electrochemical sensors

## Abstract

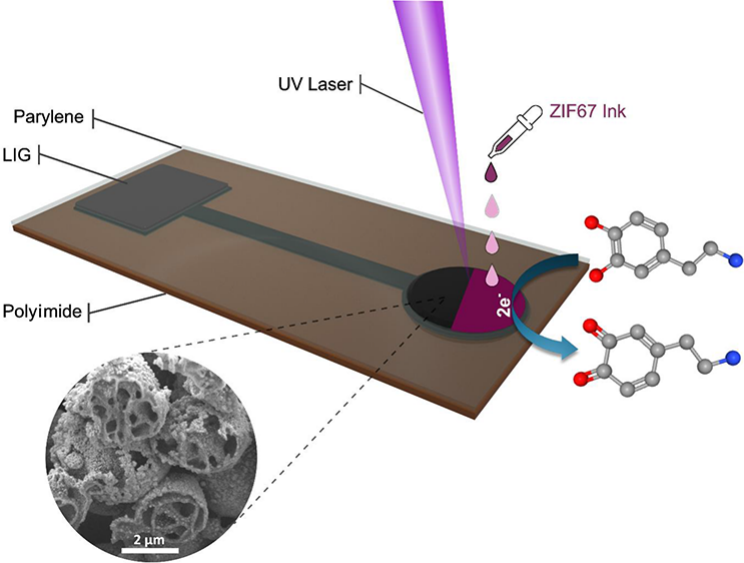

The successful development of a metal–organic framework (MOF)-derived Co/Co_3_O_4_/C core–shell composite integrated into laser-induced graphitic (LIG) carbon electrodes for electrochemical sensing is reported. The sensors are fabricated via a direct laser scribing technique using a UV laser (355 nm wavelength) to induce the photothermolysis of rationally selected ZIF-67 into the LIG matrix. Electrochemical characterization reveals that the incorporation of the laser-scribed ZIF-67-derived composite on the electrode surface reduces the impedance more than 100 times compared with bare LIG sensors. Comprehensive morphological, structural, and chemical analyses confirm the formation of porous LIG from the laser irradiation of polyimide, while the LIG+ZIF-67-derived composites feature size-controlled and uniformly distributed Co/Co_3_O_4_ core/shell nanoparticles (NPs) in the semihollow wasp-nest-like carbon matrix from photothermal decomposition of ZIF-67, embedded within the LIG electrode area. The high surface area and porosity of this ZIF-67-derived nitrogen-rich carbon facilitate charge transfer processes, whereas size-controlled Co/Co_3_O_4_ core/shell NPs offer accessible electrochemical active sites, making these LIG+ZIF-67-derived composite-based sensors promising materials for applications requiring high charge injection capability and low electrode/electrolyte interface impedance. The PI+Z67_L_ sensor exhibited a 400 times higher specific capacitance (2.4 mF cm^–2^) compared to the PI_L_ sensor (6 μF cm^–2^). This laser scribing approach enables the rapid and cost-effective fabrication of high-performance electrochemical sensors enhanced by the integration of tailored MOF-derived composites.

## Introduction

Electrochemical sensors have been widely investigated as useful tools in many applications, such as bioelectronics,^1^ in vitro diagnostics, wearables,^2^ and gas monitoring.^3^ Carbon-based materials have attracted attention in sensor fabrication due to their chemical stability, biocompatibility, versatility, and cost-effectiveness.^4,5^ Electrochemical sensors generally benefit from large effective surface areas and electrochemically stable materials.^6,7^ Thus, porous carbon-based materials are good candidates for electrode materials and have been widely used in different forms, such as glassy carbon^8,9^ and carbon nanotubes.^10,11^ Among these, one of the most promising classes of materials due to its simplicity, controllability, and cost-effectiveness is laser-induced graphene (LIG), a three-dimensional porous graphene-based material.^12,13^ LIG is obtained by laser heating a carbon-rich insulating precursor (such as a polymer film,^14^ wood,^15^ or even bread^16^) into a conductive layer according to a desired pattern. The resulting materials exhibit various degrees of electrical performance depending on the parameters, environmental conditions, and precursors.^17,18^ The most widely used precursor is polyimide (PI) due to its commercial availability, excellent mechanical and thermal properties, and good conductivity of the resulting LIG.^19−24^ However, the LIG-based sensing devices suffer from limited parameter variability, subsequently narrowing their scope for the high-performance and simultaneous detection of multiple analytes. Therefore, tuning the properties of PI-based LIG with additional materials is highly desirable.^25^

Metal–organic frameworks (MOFs) are a class of hybrid materials consisting of metal clusters or metal ions coordinated by organic ligands. MOFs are known for their rationally designed porous structures.^26−28^ The large surface areas, tunable morphologies, and extensive possibilities of functionalization of MOFs make them ideal sacrificial templates to thermally derive metal compound/carbon composite materials with high surface area and porosity.^29^ Recently, laser scribing has been used to induce the thermal decomposition of MOFs.^30−36^ Contrary to the conventional oven-based pyrolysis techniques, the photothermolysis of surface-immobilized MOFs allows for localized rapid heating and subsequent rapid cooling in a controlled fashion, offering immense possibilities of direct processing and transforming MOF (thin) films into desired composite materials on heat-sensitive substrates (such as organic polymers).^37,38^ Specifically, the use of pulsed-wave lasers has demonstrated enhanced control over thermal cycles, enabling precise material transformations and microstructure tailoring, which could inspire further innovations in MOF-derived composites.^39^ The photothermolysis undergoes three following processes: (i) efficient laser light absorption by MOFs, (ii) the generation of a local reducing atmosphere with instant heating and cooling, and (iii) carbonization of organic linkers and reduction of metal nodes to form metal nanoparticles (NPs).^37^ The laser-scribed MOF derivatives offer the advantage of precise particle size control and their subsequent distribution within the porous carbon matrix.^30^

Commonly, IR laser sources, for example, CO_2_ lasers with 10.6 μm wavelength, are employed for the fabrication of LIG^40−43^; however, very few reports have recently focused on UV laser scribing.^44^ One of these studies compared IR and UV LIG from polyimide and found that, albeit different in the density of graphene edges and pores, both films present comparable microstructures, chemical composition, and electron transfer kinetics.^45^ Another important aspect for the fabrication of a sensor is the insulation of conductor traces, which is required in most electrochemical sensing applications to provide electrical isolation from adjacent interconnection lines and analytical solutions.^46^ Herein, we aim to integrate MOF structures into LIG electrodes to improve the performance of electrochemical sensing devices. Though there are very few preliminary reports available on laser-scribed ZIF-67, this study offers in-depth elucidation of photothermolysis of ZIF-67 under an ambient atmosphere. We explored the UV-laser interaction with Co-based ZIF-67 and its subsequent decomposition and formation of core/shell Co/Co_3_O_4_ NPs. ZIF-67 as a precursor was chosen due to its high cobalt content, redox-active and mechanochemically stable Co species, inherent N and Co atoms acting as self-catalysts to form highly conducting graphitic carbon, tunable particle size, facile (green) synthesis, and scalability.

Upon exposure to high-energy photon pulses at 355 nm, the ZIF-67 framework, with its suitable optical bandgap, efficiently absorbs the incident UV laser light, inducing rapid localized heating and converting the organic linker into carbonaceous species. This highly concentrated and localized laser energy in the presence of these reductive carbonaceous species reduces metal nodes into size-controlled and well-distributed metal NPs.^37,47^ We provide laser parameter optimization for lower sheet resistance and the highest electrical conductivity. Further, we investigate the performance difference between bare LIG sensors (PI-parylene-C-derived carbon) (labeled as PI_L_ sensors) and LIG-ZIF-67 sensors (ZIF-67 ink coated on PI, labeled as PI+Z67_L_ sensors), both of which are fabricated by direct laser scribing. Detailed material characterizations reveal (contrary to the common understanding of laser metallurgy of Co-MOFs) a highly porous semihollow wasp-nest-like carbonaceous composite material with predominantly Co NPs coated with a thin shell of Co_3_O_4_ which are embedded in a nitrogen-rich porous carbon. The electrochemical tests confirmed that PI+Z67_L_ sensors exhibit a hundred times lower impedance compared to the PI_L_ sensors. This approach highlights the potential for applications requiring a high charge injection capability and low impedance at the electrode/electrolyte interface.

## Results and Discussion

### Material Characterization

**Figure 1:**
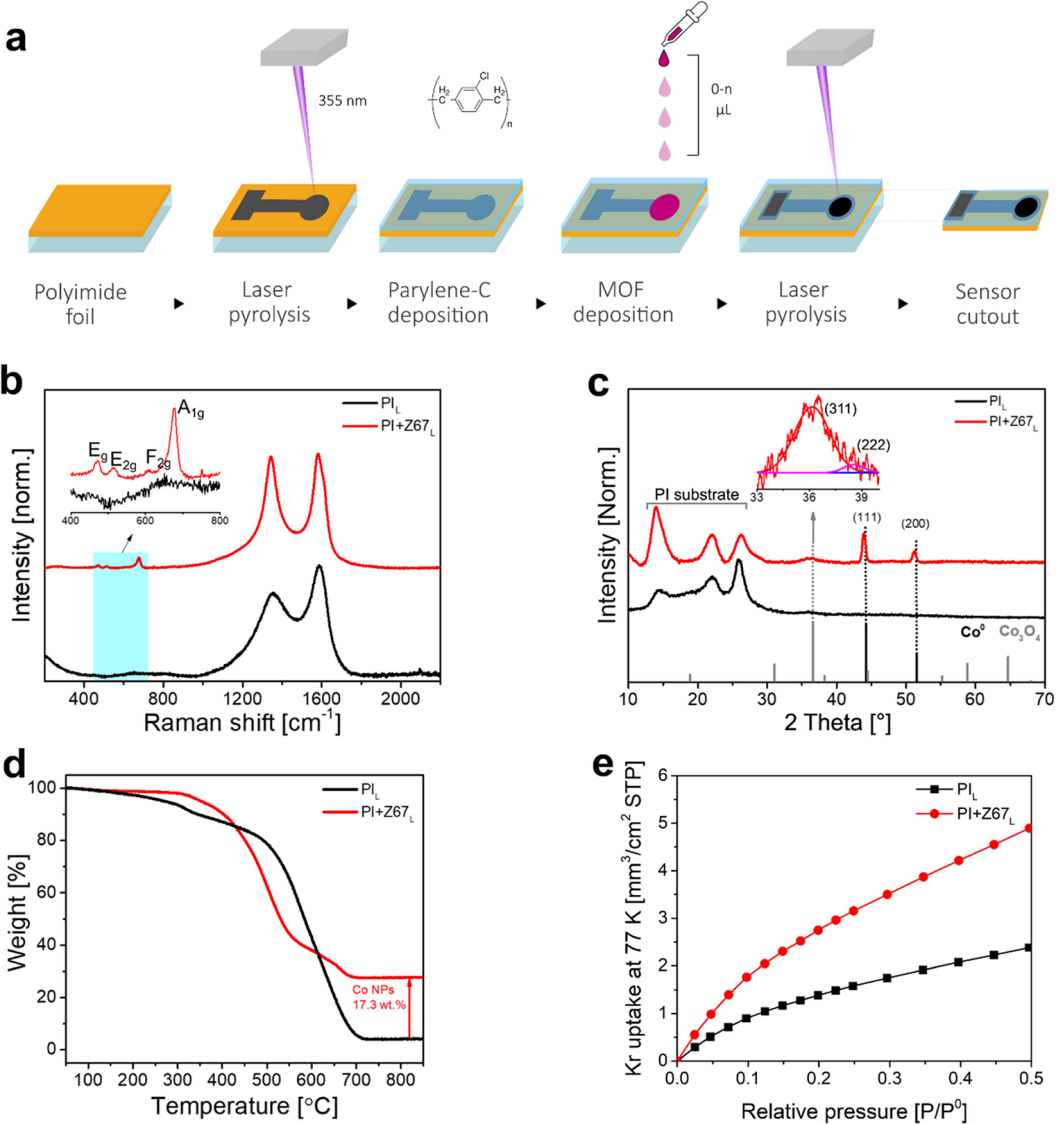
(a) Schematic representation of the fabrication steps of the laser-scribed ZIF-67-coated polyimide-based electrochemical sensor. (b) Raman spectra, (c) GI-XRD, (d) TGA, and (e) BET surface area (by Kr adsorption) of laser-scribed PI_L_ and PI+Z67_L_.

The LIG carbon was fabricated via laser direct writing on a PI-parylene-C film substrate (PI_L_ sensor). The devices were insulated by a 5 μm-thick parylene-C layer. Subsequently, for the ZIF-67-coated-PI-based sensors (PI+Z67_L_ sensor), a drop-casting method was used to apply the ZIF-67 solution onto the sensing area (electrode) of PI, followed by photothermolysis by a UV laser. The optimized parameters are summarized in Figure S1 and were chosen to minimize the sheet resistance and maximize the structural stability. An overview of all of the fabrication steps is shown in Figure 1a. The UV–vis absorption spectrum of ZIF-67 (Figure S2) confirms the good light absorption in the UV region by the imidazole linker. As mentioned above, upon exposure to the 355 nm pulsed UV laser (at optimal ns laser power, pulse duration, scan speed, defocus, and line pitch), the ZIF-67 framework undergoes a rapid sequence of events. The high-energy incident photons interact with the Co–N coordination environment and organic linkers, promoting electronic excitation within the metal–ligand complexes. These excited states rapidly relax via electron–electron scattering, transferring energy into the lattice and causing ultrafast electron–phonon coupling. The resultant localized heating drives bond cleavage in the organic linkers, initiating carbonization of the imidazole linker and the release of volatile species (CO_*x*_ and NO_*x*_). Concurrently, ns-scale laser-induced rapid heating and rapid cooling cause cobalt nodes to undergo partial reduction due to both the elevated temperature and the reactive intermediates (formed by imidazole linker decomposition) which leads to the formation of very small NPs. The self-limiting process of rapid cooling prevents extensive particle coalescence. Consequently, size-controlled Co NPs are formed and partially oxidized to Co_3_O_4_ on the surface due to ambient oxygen exposure, resulting in a core–shell structure. These steps collectively produce a uniform composite of Co/Co_3_O_4_ embedded in a nitrogen-doped porous carbon matrix, as the ns-time scale pulses allow precise spatiotemporal control over the photothermolysis.^37,47^

Raman spectra (Figure 1b, representing optimal laser parameters) confirmed the successful graphitization of PI_L_ and PI+Z67_L_ by UV laser scribing. The characteristic peaks of the D band (corresponding to lattice vibrations of graphite due to disorders, A_1g_ symmetry) and G band (representing planar sp^2^ bonded atoms in graphene/graphite, E_2g_ symmetry) appeared around 1350 and 1587 cm^–1^ in PI_L_, respectively, with an *I*_D_/*I*_G_ ratio of 0.76.^48,49^ However, in sample PI+Z67_L_, the D and G bands slightly shifted to 1343 and 1580 cm^–1^, respectively, with an *I*_D_/*I*_G_ ratio of 0.96. The red shift in D and G bands and increased *I*_D_/*I*_G_ ratio can be attributed to the presence of nitrogen-rich carbon due to the photothermolysis of ZIF-67.^50−52^ The effect of laser parameters on the nature of carbon from PI only and PI+ZIF-67 was further investigated by preparing a series of samples under varying laser speeds from 1 to 7 mm s^–1^. As shown in Figure S3, in PI_L_ only, a slower laser speed (longer exposure under air) resulted in an increased D band corresponding to the formation of a relatively higher number of defects. Also, the broadening of the D band peak with slower laser speed indicated more strain on the graphitic carbon because of higher defect density. Instead, in sample PI+Z67_L_, a slower laser speed resulted in higher graphitization (with a smaller *I*_D_/*I*_G_ ratio), a decreased D band intensity (Figure S4), and a narrower bandwidth. This result is plausible because the sample contains a thick layer of ZIF-67 deposited on PI, which is carbonized with a UV laser in an ambient atmosphere. Slower scan speeds lead to increased oxidization of the imidazole linker in ZIF-67-derived carbon, causing a release of N in the form of NO_*x*_ species and decreasing the overall N content in the sample. Moreover, the presence of Co in this sample also promotes the graphitization of carbon. In PI+Z67_L_, the presence of Raman peaks at 471 (E_g_), 515 (E_2g_), 609 (F_2g_), and 678 (A_1g_) cm^–1^ corresponds to the formation of Co/Co_3_O_4_.^53^ These results are in good agreement with ZIF-67-derived Co/CoO_*x*_/C powder (Figure S5) via conventional oven-based bulk pyrolysis (labeled as Z67_O_). Figure S6 shows the Raman spectra of pristine ZIF-67, confirming its complete carbonization under a UV laser.

The crystalline structure of laser-scribed ZIF-67 was further confirmed by grazing incident angle X-ray diffraction (GI-XRD) patterns. As shown in Figure 1c, the reflections of metallic Co are observed at 2θ of 43.9° (111) and 51.2° (200). Two minor peaks (inset in Figure 1b) at 2θ of 36.1° (311) and 2θ of 38.5° (222) confirm the presence of a trace amount of Co_3_O_4_. Conventionally, Co-MOF-derived carbon composites under an inert atmosphere form metallic Co due to its reduction potential (−0.27 V) (Figure S7), where an additional heating step is required to (partially) oxidize Co into CoO_*x*_.^54,55^ On the other hand, sintering of Co-MOF under an ambient atmosphere fully oxidizes Co metal nodes into CoO_*x*_, as well as organic linkers into CO_2_, H_2_O, and NO_*x*_. In this laser scribing technique, even under an ambient atmosphere, most of the Co is still present as metallic Co with a small amount of Co_3_O_4_ in the form of Co/Co_3_O_4_ preferably as a core/shell structure, which is uniformly distributed in a nitrogen-rich electrically conducting carbon matrix. It can be assumed that during laser scribing, the MOFs undergo very rapid heating and cooling (time scale of nanoseconds) compared to the conventional oven pyrolysis (time scale of hours), which prevents the metal species as well as carbon from complete oxidization.^34,56^ However, the presence of atmospheric oxygen as well as the (intrinsic) molecular oxygen present in MOFs (as a precursor) partially oxidizes the surface of Co to form a shell of Co_3_O_4_. It is worth noting that during the laser scribing of ZIF-67, the Co species also react with halogen species (chlorine present in the passivation layer of parylene-C and fluorine in Nafion binder), forming a trace amount of CoCl_2_ and CoF_2_ (Figure S7). However, upon washing the prepared samples with deionized water, these metal halides were easily removed due to their excellent water solubility (confirmed by XRD in Figure S7 and XPS in Figures S8 and S9).

A comparison of XRD patterns of laser-scribed ZIF-67 (PI+Z67_L_) and oven-pyrolyzed ZIF-67 powder (Z67_O_) (Figure S10) confirmed that the XRD reflection of Co_3_O_4_ (36.1°) is not present in the Z67_O_ sample. Moreover, the XRD reflections of metallic Co in PI+Z67_L_ are broadened with a shift toward lower 2θ (inset in Figure S10) compared to the sample Z67_O_ which clearly indicates that laser-scribed samples exhibit smaller particle sizes. As mentioned above, the formation of smaller particles with a core/shell structure of Co/Co_3_O_4_ in PI+Z67_L_ can be ascribed to the rapid laser heating and subsequent cooling, which prevents the NPs from aggregating and sintering into larger particles.^34^ This was further investigated by XPS, high-resolution transmission electron microscopy (HR-TEM), high-angle annular dark field (HAADF) scanning transmission electron microscopy (STEM), and energy-dispersive X-ray (EDX) STEM elemental mapping.

The amount of Co NPs in PI+Z67_L_ was quantified by thermogravimetric analysis (TGA) under synthetic air (SA). As shown in Figure 1d, PI_L_ (which is only graphitic carbon) fully oxidized into CO_2_ between 450 and 750 °C, leaving only a trace of impurities. However, in PI+Z67_L_, the major weight loss started above 350 °C due to the formation of NO_*x*_ and CO_2_ (from ZIF-67-derived nitrogen-rich carbon). An overall weight loss of 72.3% was observed up to 750 °C. The residual mass corresponds to Co_3_O_4_. The wt % of Co NPs only in PI+Z67_L_ was calculated to be 17.3 wt %. However, in oven-pyrolyzed ZIF-67 (Z67_O_) (Figure S10), the amount of Co NPs is 49.8 wt %. A relatively small Co content (wt %) in PI+Z67_L_ is due to the fact that this sample contains a larger amount of carbon contributed by pyrolyzed PI and imidazole linker of ZIF-67.

The porosity of the PI_L_ (with and without the 5 μm-thick parylene-C passivation layer) and PI+Z67_L_ samples was studied using Krypton adsorption at 77K (Figure 1e) and compared to a reference PI sample.^57^ As expected, the pristine PI substrate exhibits no porosity (Figure S11); however, a significant increase in Krypton uptake was observed in laser-scribed PI_L_ samples (Figure 1e), which indicates the formation of porosity during the graphitization process. It is worth noting that the parylene-C deposition as a passivation layer seems to have a negligible contribution to the overall porosity of the resulting porous carbon network. The laser-scribed ZIF-67-deposited substrate (PI+Z67_L_) led to further Krypton uptake, resulting in a significant increase in the surface area. Using multipoint Brunauer–Emmett–Teller (BET) analysis on the collected Krypton adsorption isotherms, the BET surface areas for the PI_L_ and PIL+Z67_L_ samples were estimated to be 86 and 177 m^2^/m^2^, respectively.

**Figure 2:**
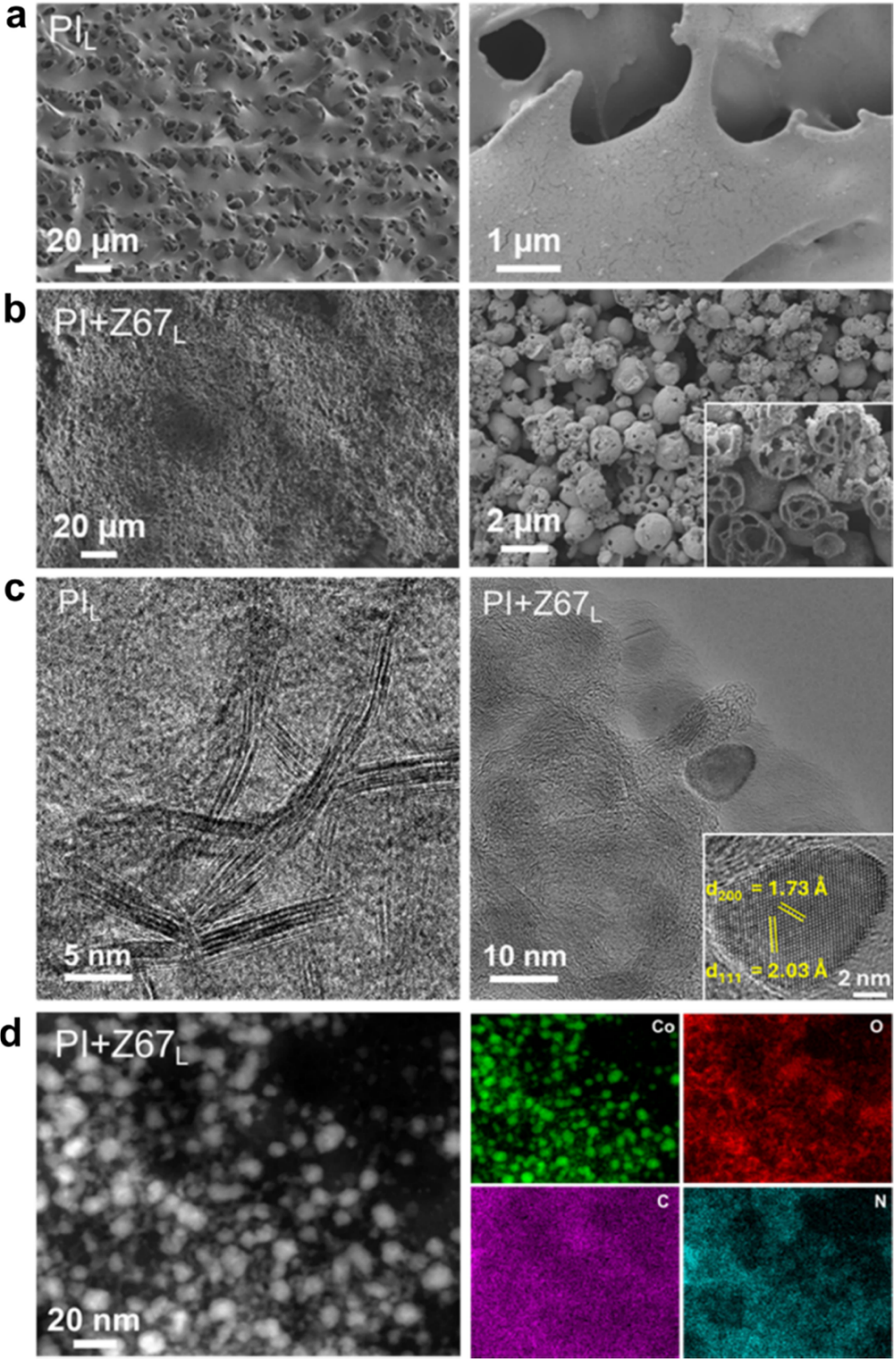
SEM images of (a) PI_L_ and (b) PI+Z67_L_. The inset in (b) (right side) shows the FIB-SEM of laser-scribed ZIF-67. The inside of the particles reveals randomized semihollow wasp-nest-like structures of carbon with Co/Co_3_O_4_ NPs. (c) HR-TEM images of PI_L_ (left) and PI+Z67_L_ (right). The inset in (c) confirms the crystal structure of the highlighted HR-TEM lattice image of a selected Co NP. (d) HAADF-STEM (left) and STEM-EDX elemental maps (right) of selected PI+Z67_L_ show the uniform distribution of well-defined Co NPs in N- and O-rich graphitic carbon.

The scanning electron micrographs of PI_L_ confirmed the graphitization of the polyimide/parylene-C substrate. The selected images (Figure 2a) of PI_L_ show a macroporous interconnected graphitic carbon foam-like structure. Laser parameters play a crucial role in the morphology, degree of graphitization (Raman spectra, Figures 1 and S4), and electrical properties (sheet resistance, Figure S1) of laser-scribed graphite. The SEM images of selected samples (Figure S12) clearly indicate that a slower laser speed forms foamy surfaces with relatively open structures, whereas samples prepared with a faster laser speed result in relatively smoother surfaces with less roughness. The ZIF-67-coated polyimide substrate (PI+Z67_L_) shows complete carbonization of ZIF-67 as well as PI. Figure 2b shows uniformly coated laser-scribed ZIF-67-derived Co/Co_3_O_4_/C NPs. To further investigate the internal morphology of ZIF-67-derived carbon, focused-ion beam (FIB) SEM was performed. It is interesting to observe that the laser scribing of ZIF-67 transforms polyhedral solid structures (Figure S13, FIB-SEM of pristine ZIF-67) into randomized semihollow wasp-nest-like structures (inset in Figure 2b; right). HR-TEM images revealed the nature of the laser-scribed polyimide and ZIF-67. The laser scribing of the polyimide substrate (PI_L_) forms a stacked sheet-like graphitic carbon structure (Figure 2c; left), whereas ZIF-67 converts into nitrogen-doped defect-rich porous carbon. The Co species are converted into crystalline Co/Co_3_O_4_ core–shell structures with high crystallinity and enveloped in onion-ring-like carbon layers (Figure 2c; right). The crystal structure of NPs is confirmed by measuring the lattice spacing of (200) and (111) Bragg planes as 1.73 and 2.03 Å, respectively, which is in good agreement with the crystal structure of Co (Figure 2c; right, inset). The bright particle contrast in the HAADF-STEM image, shown in Figure 2d (left), confirms a narrow particle size distribution of Co/Co_3_O_4_ with a particle size ranging from 5 to 15 nm. Elemental STEM-EDX mapping (Figure 2d, right) further confirmed the uniform distribution of Co NPs as well as N and O throughout the carbon matrix. A high-resolution STEM-EDX mapping and intensity line scan (Figure S14) of a selected Co NP further confirmed the presence of a thin oxide shell on the surface of the Co NP. As a comparison, HAADF-STEM images (Figure S15) and TEM images as well as EDX mapping of Z67_O_ (oven-pyrolyzed ZIF-67) (Figure S16) reveal the formation of relatively large Co NPs (20–60 nm) caused by coalescing under a slower heating and cooling process.

**Figure 3:**
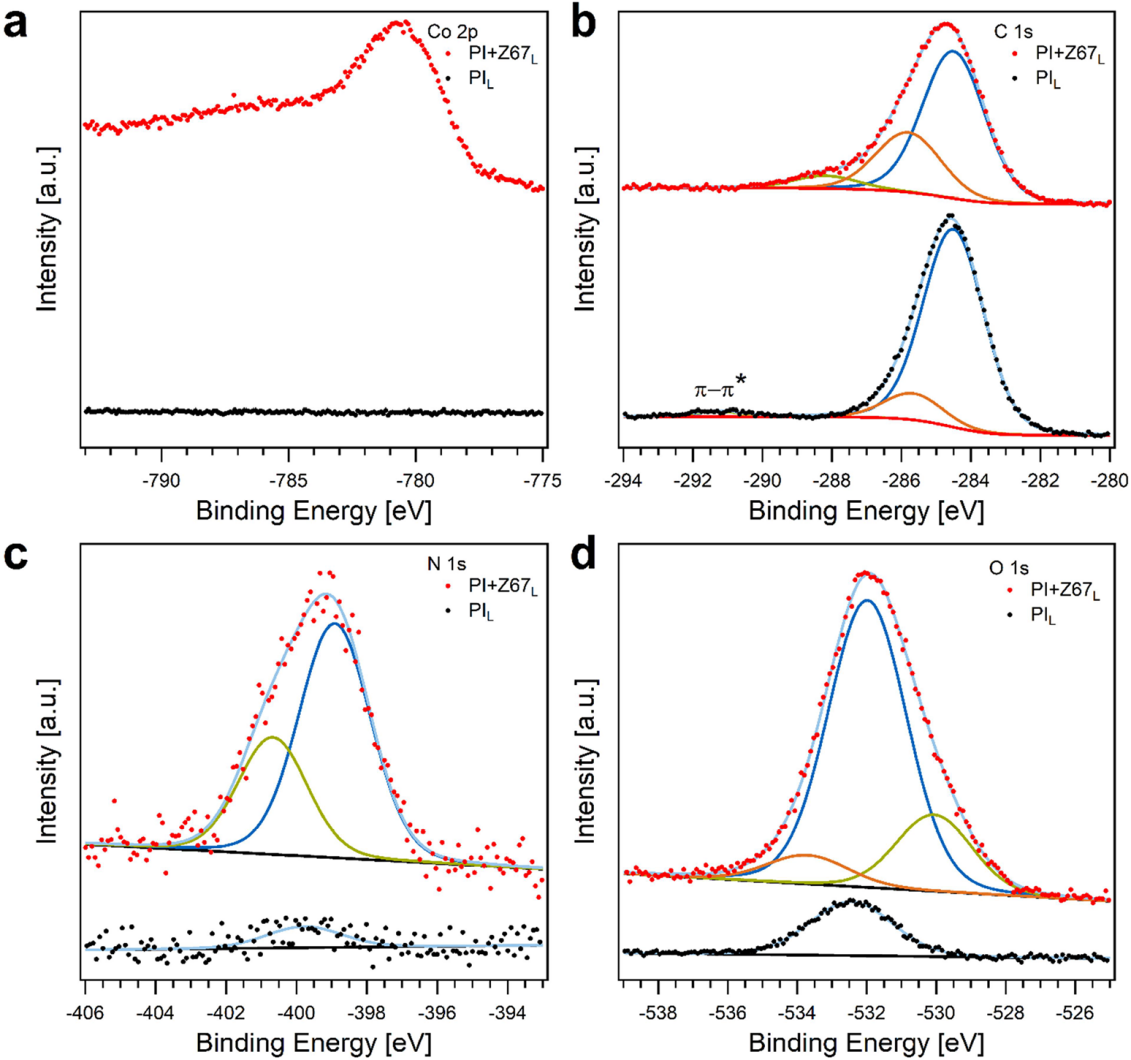
XPS spectra of (a) Co 2p, (b) C 1s, (c) N 1s, and (d) O 1s of laser-scribed PI_L_ and PI+Z67_L_.

X-ray photoelectron spectroscopy (XPS) of these samples confirmed the presence of Co on the surface of PI+Z67_L_ (Figure 3a). The binding energy of the main feature of the Co 2p_3/2_ core level at 780.3 eV, as well as the presence of a broad satellite peak centered around 786 eV, shows that Co is predominately present in its oxidic form.^58^ The deviation from the results obtained by XRD, Raman spectroscopy, and electron microscopies proving the presence of metallic Co NPs originates from the surface sensitivity of XPS; considering an inelastic mean free path of 1.2–1.4 nm of photoelectrons emitted from the Co 2p core level (*E*_kin_ ≈ 705 eV) and a thickness of 2.5 nm of the native oxide on bulk Co, one would expect a metallic component of less than 10% of the total peak area.^59,60^ Such a metallic component cannot be resolved due to the spectrally broad Co 2p core level, which might result from a variety of different chemical environments. This is expected considering the different accessible cobalt oxides and hydroxides as well as the size distribution of the Co NPs. Note that the oxidic Co on the surface of PI+Z67_L_ might be reduced to the metallic state at the potential applied in electrochemical sensing since metallic Co is the thermodynamically stable species.

The deconvoluted spectra of C 1s (Figure 3b) of PI_L_ and PI+Z67_L_ exhibit peaks at 284.5 and 285.8 eV, corresponding to C–C and C–O/C–N, respectively.^52,61^ An additional component at 288.2 eV is observed for PI+Z67_L_, which can be attributed to the C=O/COOH groups. Besides the appearance of a C=O/COOH component, the C–O/C–N peak in PI+Z67_L_ is relatively strong with respect to that in PI_L_, which is due to the presence of ZIF-67-derived nitrogen-rich carbon (Figure 3c) and an increased amount of oxygen bound in an organic matrix (Figure 3d). These results are in good agreement with Raman spectra (Figures 1a, S3, and S4). The additional satellite at 291.3 eV observed for PI_L_ is characteristic of π–π* transitions in graphitic systems.^62^ The absence of such a satellite peak for PI+Z67_L_ might be explained by the surface sensitivity of XPS. The high amount of Co NPs within the surface-near region can disturb the π-system of the surrounding carbon matrix.

In PI+Z67_L_, the N 1s core level can be deconvoluted by two peaks at 398.9 and 400.7 eV (Figure 3c), which are characteristic of pyridinic N and pyrrolic N, respectively, and go in line with the intense C–O/C–N component in the C 1s core level of PI+Z67_L_.^53,63^ The photoemission intensity recorded from the N 1s core level of PI_L_ is ∼15 times lower compared to that of PI+Z67_L_. The single component located at 399.8 eV is in good agreement with N in PI.^64^ Similar to the N 1s core levels, the O 1s peak in PI+Z67_L_ is more intense by a factor of ∼7 in comparison to PI_L_. Besides the component for oxygen in Co oxides at 530.1 eV,^58^ which is present in PI+Z67_L_ only, the increase in O 1s intensity originates from the main component around 532 eV. As the intensity of the C–O/C–N and C=O/COOH components increases accordingly, it can be concluded that the surface of PI+Z67_L_ consists of more oxygen-containing functional groups. The minor O 1s component at a binding energy of ∼533.7 eV is attributed to water adsorbed on the PI+Z67_L_ surface.

It is interesting to note that an additional peak of Cl 2p was observed in both samples (Figure S8). In PI_L_, a single spin–orbit-split component appeared at a Cl 2p_3/2_ binding energy of 200.2 eV, which corresponds to the presence of C–Cl. However, in PI+Z67_L_, an additional feature at 198.1 eV (Cl 2p_3/2_) was observed, which indicates the formation of a Co-related Cl species.^65^ As CoCl_2_ would have been removed during sample preparation involving rinsing with water, it seems plausible that these Cl species act as anchoring sites for the Co NPs in the graphitic carbon matrix. The origin of the Cl 2p peaks is from the chlorine species present in parylene-C used as a passivation layer. Moreover, a low-intensity single peak of F 1s at 689 eV (Figure S9) corresponds to the Nafion residues, used as a binder to immobilize the ZIF-67 ink on the PI substrate, which is easily removed by washing the sample with deionized water.

### Electrochemical Measurements and Analysis

**Figure 4:**
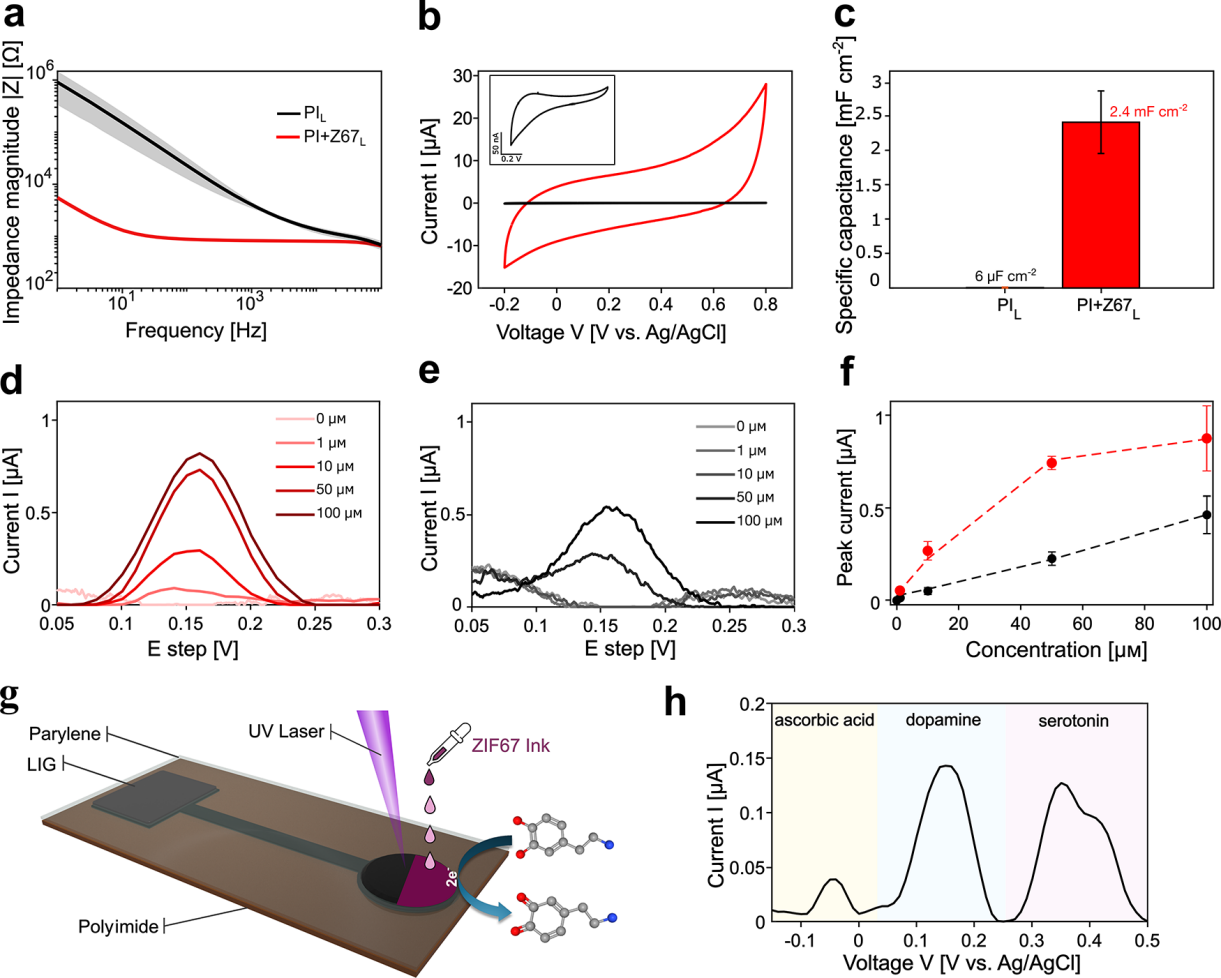
(a) Impedance spectroscopy of PI_L_ (black) and PI+Z67_L_ sensors (red) and (b) corresponding cyclic voltammograms recorded in a PBS solution. The inset shows a highlighted CV for the PI_L_ sensor. The third cycle is plotted for the CVs. (c) Specific capacitance for each sensor is also shown. (d) DPV response for dopamine (ranging from 0 to 100 μM) using the PI+Z67_L_ 4-layer sensor in PBS. (e) DPV response for dopamine (ranging from 0 to 100 μM) using the PI_L_ sensor in PBS. (f) Corresponding calibration plot showing peak current amplitude versus concentration. (g) Schematic representation of the DA detection mechanism by laser-scribed PI+Z67_L_ electrodes. (h) DPV response of the PI+Z67_L_ sensor for the detection of dopamine (DA) in the presence of the typical interfering analytes: ascorbic acid (AA) and serotonin (5-HT). The concentrations of the analytes are as follows: 200 μM DA, 200 μM 5-HT, and 500 μM AA in 10 mL PBS.

The electrochemical behavior of both PI_L_ and PI+Z67_L_ sensors was analyzed using electrochemical impedance spectroscopy (EIS) and cyclic voltammetry (CV). A pure PI_L_ sensor was used as a reference to evaluate the electrochemical performance of the PI+Z67_L_ sensors. The Bode plots in Figure 4a show that the average impedance magnitude of the PI+Z67_L_ sensor decreases 100 times compared to that of the PI_L_ sensor, indicating that the electrochemical performance of the PI+Z67_L_ electrode is improved when the ZIF-67 ink is deposited on the sensing area followed by one-step laser scribing. The CV measurements (Figure 4b) confirm these results, with the PI+Z67_L_ electrode (fabricated under optimized laser-scribing parameters) showing the best performance due to the provision of higher surface areas, more electrocatalytic active sites, and improved charge transfer at the electrode–electrolyte interface. Figure 4c shows a comparison of the specific capacitance of the prepared sensors. The capacitance (*C*) of the PI_L_ electrodes was calculated from the difference of the anodic and cathodic current (*i*_a_ and *i*_c_, respectively) at the scanning rate (*v*) of 100 mV s^–1^ with the relationship at a working electrode potential of 0.3 V. The potential is chosen to avoid the possible interference of Faradaic reactions such as oxygen reduction.

The specific capacitance is calculated by dividing the capacitance by the geometric area of the sensor. As shown, the specific capacitance of the PI_L_ sensor is about 6 μF cm^–2^, while the specific capacitance of the PI+Z67_L_ sensor is around 2.4 mF cm^–2^, indicating a 400 times increase in capacitance due to the presence of additional carbonaceous species on sensing area contributed by laser-scribed ZIF-67. This behavior is expected since pyrolyzed ZIF-67 typically offers higher surface areas and well-developed porosity. When combined with laser-scribed graphitic carbon, this can significantly increase the overall surface area available for charge storage, leading to higher capacitances. In addition, the interconnected pore structure of the laser-scribed ZIF-67 improves ion accessibility and transport, contributing to increased capacitance.

The analytical performance of the PI+Z67_L_ electrode for the detection of dopamine (DA) as a model system was investigated using the differential pulse voltammetry (DPV) technique due to its low charge contribution to the background current and high current response. The anodic peak current of DA at concentrations between 1 and 100 μM measured with the PI+Z67_L_ sensors is shown in Figure 4d. The maximum anodic peak of DA occurred at +0.15 V and increased with the DA concentration, as shown in Figure 4f (red trace). However, at higher DA concentrations, the trend indicates that the electrode surface becomes saturated, limiting the detection capabilities. Figure 4e shows the same experiment performed with the PI_L_ sensor. The results show that when pyrolyzed ZIF-67 is not present on the electrode area, the sensitivity of the sensor to dopamine decreases. This behavior is also shown in Figure 4f where we can see the comparison between the two trends of peak current vs concentration, from the PI_L_ and PI+Z67_L_ sensors.

The EC detection mechanism of DA is well-established for carbonaceous composite electrode materials.^66^ Specifically, DA sensing has been previously shown using oven-pyrolyzed carbonaceous electrodes within comparable concentration ranges,^67^ and some studies report a linear response to DA within the 100 nM–1 μM range.^68^ In this study, we did not optimize for the limit of detection but aimed at characterizing the DA sensing performance of the photothermally derived ZIF-67 composites (PI+Z67_L_) in comparison to polyimide-derived graphitic carbon (PI_L_). DA molecules adsorb onto the graphitic carbon due to its available surface area and the π–π interactions. Although graphitic carbon (PI_L_) is electrically conductive and active in DA oxidation, its lower surface area and limited charge transfer capacity result in poor electrochemical (EC) performance. However, using laser-scribed ZIF-67-derived nitrogen-rich porous carbon decorated with Co/Co_3_O_4_ NPs as an electrode material (PI+Z67_L_) offers superior electrical conductivity and electrocatalytically active sites. The improved adsorption of DA molecules on high surface area nitrogen-rich porous carbon and its proximity to Co/Co_3_O_4_ NPs enhance the possibility of interaction and subsequent electron transfer for dopamine oxidation. The oxidation of DA takes place at the electrode surface, forming dopamine-*o*-quinone and concurrently releasing protons and electrons. The generated electrons correspond to the detected current, proportional to the concentration of DA. Figure 4g illustrates this mechanism. Along with the porous carbon, Co/Co_3_O_4_ plays a crucial catalytic role, facilitating the electron transfer and enhancing reaction kinetics, making the oxidation of dopamine more efficient.^66,69,70^ The EC oxidation reaction of DA can be described as follows:
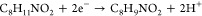


Dopamine detection in biological samples is notoriously challenging due to the presence of various interfering species. Ascorbic acid (AA) and serotonin (5-HT) are two common interferents that can significantly affect the accuracy of dopamine measurements. To validate the selectivity and practical utility of the PI+Z67_L_ sensor for dopamine detection, interference tests with AA and 5-HT were performed (Figure 4h). These tests provide information on the sensor’s performance in the presence of typical interferents helping to establish its potential for real-world applications in dopamine sensing. The DPV results demonstrate the potential of the PI+Z67_L_ electrodes for dopamine sensing and analysis within certain physiologically relevant concentration ranges.

The amount of ZIF-67 precursor and its loading via ink drop casting were optimized to obtain a uniform coating, as drop casting of more than 0.2 mg of diluted ink resulted in a nonuniform sensor area profile (Figure S17). A known concentration of ZIF-67 ink was deposited dropwise with one to four repetitions of drop casting (50 μg–0.2 mg). The electrodes were coated with different amounts of ZIF-67 ink, and their subsequent coating thickness was laser-scribed under optimal laser parameters. All of these prepared electrodes were analyzed to determine their layer thickness and electrochemical response. When the ZIF-67 loading exceeded the fourth repetition (0.2 mg ZIF-67 loading), the thickness distribution of the sensing area became nonuniform (Figure S17a), which affected the mechanical stability of the coating. The electrode thickness increased proportionally with the amount of ZIF-67 deposited (Figure S17b). Figure S18 shows the electrochemical characterization for different amounts of ZIF-67 and the DPV detection of dopamine. The CV and impedance measurements of laser-scribed PI+Z67L (Figure S18a,b) show that increasing the thickness of the electrode layer leads to a decrease in impedance and a subsequent increase in current. DPV detection (Figure S18c) shows that the sensitivity of the electrode to DA increases with a higher ZIF-67 loading up to a mass loading of 0.2 mg and a layer thickness of 25 μm. Beyond this point, a further increase in the electrode layer thickness hinders dopamine detection.

This occurs because an excessively thick electrocatalyst layer adversely affects DPV results through several factors, including increasing the resistance of the layer, hindering electron transfer, and reducing the current response. In addition, diffusion limitations impede the transport of reactants and products to and from active sites, thereby reducing the electrocatalytic efficiency. This leads to the inaccessibility of the analyte across the thick layer. In our case, when the deposited ZIF-67 layer became too thick, it resulted in poor mass diffusion, which slowed the transport of analyte molecules to the electrode surface. In addition, an increase in double-layer capacitance also affected the DPV measurements. Therefore, the optimal mass loading of the ZIF-67 ink was limited to 0.2 mg, which provided a good compromise between the electrochemical performance and dopamine detection.

To compare the performance of ZIF-67-derived Co/Co_3_O_4_/C with commercially available cobalt NPs, identical electrodes were prepared either laser-pyrolyzed or nonpyrolyzed (as deposited). As shown in Figure S19, the sensors loaded only with commercial Co NPs (as deposited/nonpyrolyzed) resulted in significantly poor specific capacitance (0.39 mF cm^–2^) compared to that of PI+Z67_L_ (2.4 mF cm^–2^). This high performance of laser-pyrolyzed ZIF-67-derived composites can be attributed to the size-controlled and catalytically accessible Co/Co_3_O_4_ NPs uniformly dispersed in a N-rich graphitic carbon matrix, contributing to the increased porosity and effective surface area. In addition, a simple adhesion test (scotch tape method) was performed, and it can be concluded that PI+Z67_L_ is the most structurally stable.

### Electrochemical and Mechanical Stability

**Figure 5:**
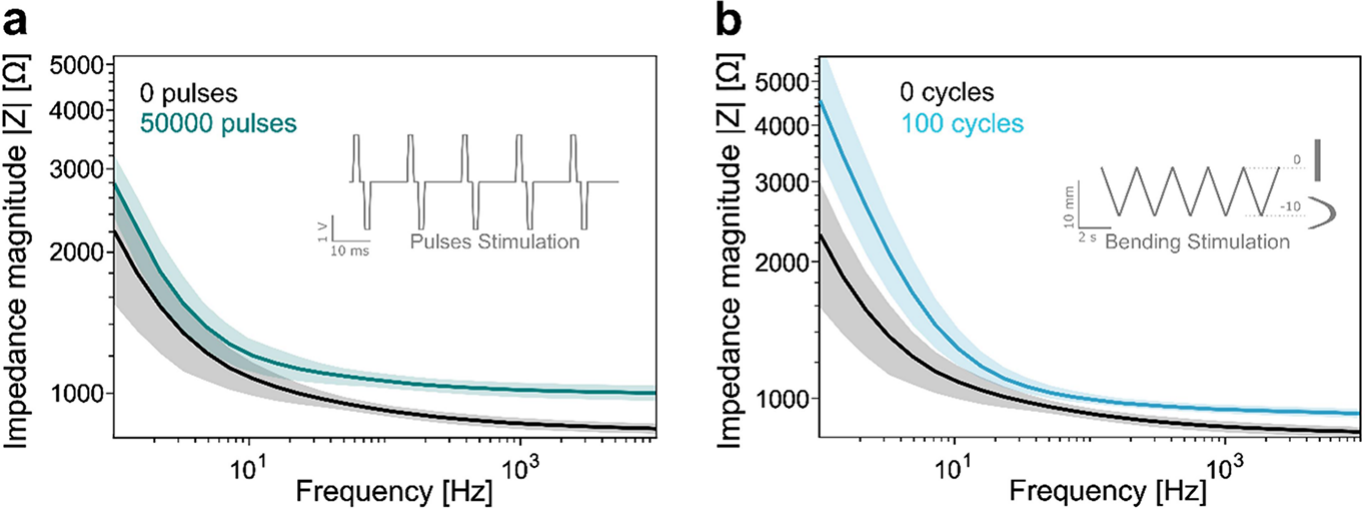
Stability tests of the PI+Z67_L_ 4-layer sensor. (a) Electrochemical stability of the PI+Z67_L_ 4-layer electrode evaluated before and after 50,000 biphasic pulses (10 V, 10 ms each). (b) Mechanical stability of the PI+Z67_L_ 4-layer electrode evaluated before and after 100 bending cycles (0 to −10 mm, 2 s each).

Electrode stability is critical to the performance and longevity of electrochemical sensors. Ensuring both electrochemical and mechanical stability helps to maintain consistent sensor responses and prevents degradation over time, which is essential for accurate and reliable measurements. The electrochemical and mechanical stability of the PI+Z67_L_ sensors was tested, as displayed in Figure 5. Figure 5a shows the change in impedance when the electrodes were subjected to 50,000 biphasic stimulation pulses of ±2 V with a duration of 1 ms per phase. The results show that the average impedance magnitude of the electrodes increased slightly after the 50,000 pulses; at 1 kHz, the magnitude of the impedance increased 1.2 times, from ≈0.8 to ≈1 kΩ. Interestingly, the changes in impedance occurred during the first 20,000 pulses reaching a stable plateau afterward (Figure S20). We attribute this behavior to the initial cleaning of the electrodes by applying stimulation pulses. The mechanical bending test results (Figure 5b) show the change in impedance when electrodes were bent at a constant speed of 10 mm min^–1^ for 100 cycles up to a bending angle of approximately 60°. Again, the results show that the average impedance magnitude of the electrodes increased slightly after 100 compression cycles. For example, at 1 kHz, the magnitude of the impedance increased 1.1 times, from ≈0.8 to 0.9 kΩ. This result is comparable to other LIG-based sensors, which show low resistance change within the 0°–50° bending interval and high stability when subjected to mechanical fatigue testing.^44,71^

The electrochemical and mechanical stability tests demonstrate the robustness of the PI+Z67_L_ sensors. Despite being subjected to 50,000 electrochemical pulses and subsequent bending, the impedance values of the electrodes showed only a minor increase. This indicates the ability of the sensor to maintain its chemical and structural integrity without undergoing significant degradation or side reactions that could compromise its performance and longevity. Furthermore, the GI-XRD (Figure S21) of the used PI+Z67_L_ sample (after EC sensing) confirmed the structural stability of Co/Co_3_O_4_/C species after EC sensing. There was no significant change observed in the crystalline structure of Co species. The HR-TEM and HAADF-STEM images and EDX elemental maps of PI+Z67_L_ (Figure S22) revealed a minor aggregation of Co species after being tested for EC sensing in a PBS environment; however, the particle sizes of Co/Co_3_O_4_ did not change. These results highlight the potential of the PI+Z67_L_ sensors for practical applications that require long-term operation and deformation that may occur during operation or handling.

## Conclusions

We have successfully fabricated a ZIF-67-coated PI-derived composite as an electrochemical sensor by photothermolysis. The proposed sensors are fabricated by UV laser scribing (355 nm) of PI to form the LIG feedlines. The device is passivated with parylene-C, a vapor-deposited polymer that conforms well to the porous, 3D structures of LIG. At the sensing sites, ZIF-67 is photothermally decomposed into the LIG matrix in a second laser scribing step to obtain a PI+Z67_L_ sensor with insulated PI_L_ feedlines. Detailed characterization of the resulting materials from the two sensors (PI_L_ and PI+Z67_L_) confirmed that the laser irradiation of polyimide under an ambient atmosphere corresponds to LIG carbon, while the PI+Z67_L_ sensors form very small sized Co/Co_3_O_4_ core/shell NPs of 5–15 nm, uniformly embedded in the semihollow wasp-nest-like porous carbon matrix in the electrode region. Electrochemical analysis of various samples reveals that the laser-scribed ZIF-67-derived composites at the electrode area exhibit more than 100 times lower impedance than the polyimide-based PI_L_ sensor. These results underscore the effectiveness of the laser scribing technique in fabricating low-impedance, high-performance electrochemical sensors in a rapid, cost-effective method. Furthermore, the presence of MOF-derived composites significantly enhances the electrochemical performance of the sensor by providing higher specific surface areas and accessible electrocatalytic active sites, facilitating charge transfer and electrochemical reactions at the electrode–electrolyte interface. We believe that by further optimizing laser parameters and modulating the chemical composition of precursor MOFs, this approach opens a new possibility of high-performing and stable fully customizable flexible electrodes. This study provides the foundation for the miniaturization of MOF-based electrochemical sensors, paving the way for multielectrode arrays for the simultaneous detection of different analytes.

## Methods

### Materials

Kapton PI with a thickness of 125 μm was used as the substrate for laser scribing in this work. The ZIF-67 was selected for this work. CV and impedance spectroscopy were performed in phosphate-buffered saline (PBS) purchased from Sigma-Aldrich (St. Louis, MO). 2-Propanol (≥99.5%) and ethanol (≥99.5%) were purchased from Carl Roth (Germany). Deionized water was produced by a water purification system (Berry Tech, Germany), and a parylene-C dimer (dichlorodi-*p*-xylylene) was obtained from Specialty Coating Systems (USA). Nafion perfluorinated resin solution at 5 wt % was purchased from Sigma-Aldrich. Dopamine hydrochloride (4-(2-aminoethyl)-1,2-benzenediol) was also purchased from Sigma-Aldrich.

### MOF Synthesis and MOF Ink Preparation

ZIF-67 was synthesized by following a reported method with some modification.^53,72^ Briefly, Co(NO_3_)_2_·6H_2_O (0.291 g, 1 mmol) and 2-methylimidazole (0.657 g, 8 mmol) were dissolved in methanol (40 mL) separately. Once fully dissolved, the Co(NO_3_)_2_·6H_2_O solution was added into the 2-methylimidazole solution under stirring and left at room temperature for 20 h. The precipitates were collected by centrifugation, followed by washing with methanol 3 times to remove residual species. The washed samples were dried at 70 °C overnight under air.

The MOF ink was prepared by mixing ZIF-67 (5 mg) in ethanol (50 μL), deionized water (50 μL), and Nafion (perfluorinated resin solution) (2.5 μL). To achieve a uniform dispersion of the ZIF-67 particles in the ink, the mixing was performed using a vortex mixer (MS2, IKA-Werke GmbH & Co. KG, Germany) operating at 2500 rpm for a few minutes. This vigorous mixing helped to break down any agglomerates and ensure that the ZIF-67 particles were well dispersed in the ethanol/water/Nafion solution to form a homogeneous ink formulation.

The ZIF-67-derived Co/C sample, labeled as Z67_O_, was obtained by pyrolyzing 500 mg of ZIF-67 in a tube furnace for 2 h under an argon atmosphere. The ramp rate was set at 5 °C min^–1^.^52^ This oven-pyrolyzed powder sample Z67_O_ was characterized directly without any further treatment.

### Sensor Fabrication

The 0.3 in. x 1 in. sensors, laser-scribed polyimide films (labeled as PI_L_) and ZIF-67-coated-parylene-passivated polyimide films (labeled as PI+Z67_L_), were fabricated with the laser scribing technique using a UV nanosecond pulsed laser scanner (MD-U1000, Keyence, Osaka, Japan). The electrode was 2 mm in diameter, and the feedline was 5 mm in length. A 125 μm-thick PI foil was fixed on a glass slide as a support and cleaned with isopropanol. The laser scribing process on PI_L_ was performed at 10% power, 7 mm s^–1^ scan speed, 400 kHz pulse frequency, 1 repetition, 1 mm defocus, and 20 μm line pitch (Figure S1). Parylene-C (5 μm) was deposited via chemical vapor deposition (PDS 2010, SCS Labcoater 2 Parylene Deposition System, Specialty Coating Systems, USA) to passivate the entire sensor surface.

To fabricate the PI+Z67_L_ sensor, the MOF ink (1 μL of ink, around 50 μg of ZIF-67) was drop-cast on each electrode area and allowed to dry in air. The drop-casting process was repeated up to four times (for a total of around 0.2 mg of ZIF-67) to observe the influence of the MOF amount on the device performance (Figures S15 and S16). No MOF ink drops were deposited on the bare PI_L_ sensors. The laser thermolysis of the ZIF-67 and the opening of the sensing areas and contact pads were performed by laser scribing (0.5% power, 7 mm s^–1^ scan speed, 400 kHz pulse frequency, 1 repetition, and 1 mm defocus; Figure S1). Finally, the sensors were cut out with the same laser (100% power, 400 mm s^–1^ scan speed, 50 kHz pulse frequency, 23 repetitions, and 0 mm defocus) and then released from the glass slide support.

### Material Characterization

The GI-XRD patterns were recorded on a PANalytical Empyrean diffractometer equipped with a PANalytical PIXcel1D detector. X-ray Cu Kα radiation (λ = 1.5406 Å) operated at 45 kV (voltage) and 40 mA (intensity) was used for the measurements. The diffractograms were recorded for 2θ angles between 10° and 70° with a position-controlled flat sample stage for thin-film measurements with a step size of 0.005° and a scan rate of 0.2° min^–1^. The incident beam was focused on the sample through a focusing beam mirror with 0.04 rad Soller slits, a 1/8° divergence slit, and a 4 mm mask. The micrographs of laser-scribed samples were recorded by a JEOL JSM-7500F field-emission scanning electron microscope (SEM). FIB coupled with SEM was performed using Bal-tec MED 020 and a scanning electron microscope (Lichtenstein, Gemini 2, Zeiss Crossbeam 550, Germany). The FIB images were acquired using the following parameters: SEM beam voltage 1 kV, working distance 5 mm; the FIB Ga beam was accelerated at 30 kV with a current of 15 nA. For the transmission electron microscopy (TEM), the samples were prepared by dispersing powders in the ethanol solution, which were then ultrasonicated for 45 min, followed by drop casting on a standard TEM Cu sample grid with holey carbon support. TEM images were acquired using Thermo Fisher Spectra Ultra, operated at 300 keV and equipped with an extra brightness field emission gun (X-FEG). The objective lens aberration correction was done before the experiment. The exposure time for acquiring a single lattice image was 1 s, with an image size of 4K × 4K and a pixel size of 0.017 nm. The HAADF-STEM images were acquired by using a convergent probe with a beam convergence angle of 21 mrad, with a dwell time of 5 μs per pixel. The camera length was 220 mm with a collection angle of 31–188 mrad. The screen current was 91 pA. The EDX mapping of elements was carried out using Thermo Fisher Spectra Ultra, operated at 300 keV and equipped with a 6-segmented Ultra-X EDX detector of a solid angle greater than 4.45 Sr. The UV–vis absorption spectrum of pristine ZIF-67 was recorded by Shimadzu UV-3600i plus.

To quantify the carbon and Co content of the electrodes, the TGA of laser-scribed PI and PI+Z67 was performed by a Mettler Toledo TGA/STA 409PC apparatus under synthetic airflow with a heating ramp of 10 °C min^–1^. The Renishaw inVia Reflex Raman System RL532C, Class 3B was employed to record the Raman spectra of all the samples. XPS spectra were recorded on a Leybold-Heraeus LHS 10 spectrometer using a nonmonochromatized Al K_α_ source (1486.7 eV). Laser-scribed polyimide samples (PI and PI_L_+Z67_L_) were fixed on a sample holder by screws, and the analyzed front was grounded. Powder samples were pressed into cavities and measured as pellets. All spectra were recorded in an ultrahigh vacuum chamber at a pressure below 2 × 10^–7^ mbar. The analyzer was operated at a constant pass energy of 100 eV leading to an energy resolution with a full width at half-maximum (fwhm) of ∼1.1 eV. The energy scale was corrected for sample charging by using the C 1s main signal at 284.5 eV. Core level spectra were deconvoluted by Voigt functions after linear or Shirley background subtraction.

Krypton adsorption isotherms were measured by using a Micromeritics 3Flex physisorption instrument at 77 K up to a relative pressure (*P*/*P*^0^) of 0.65. Before measuring, the samples were evacuated at 120 °C under vacuum for 12 h. The Kr adsorption isotherms collected were interpreted using multipoint BET analysis for surface area determination over the 0.06–0.20 relative pressure (*P*/*P^0^*) range and with a Kr cross-sectional area of 0.210 nm^2^.

### Electrochemical Characterization

All electrochemical measurements were performed using a VSP-300 potentiostat (BioLogic Instruments, France) in a three-electrode setup, with the LIG or LIG/MOF sensor as the working electrode, an Ag/AgCl (3 M NaCl) reference electrode, and a coiled platinum wire as the counter electrode. EIS and CV were used to determine the electrochemical performance of the sensors.

First, a CV step (±1.5 V at 500 mV s^–1^) was performed to clean the electrode surfaces from possible residues of the laser process. This step was also used to identify the electrochemical window of water. Impedance spectroscopy data were obtained between 1 and 10^5^ Hz with a signal of 10 V_rms_, while CV curves were obtained with a potential window from −0.2 to 0.8 V at a scan rate of 200 mV s^–1^.

The electrochemical measurements by CV and EIS were all performed using a PBS electrolyte. DPV was used for dopamine detection (pulse amplitude *E*_pulse_ 10 mV, pulse length *t*_pulse_ 10 ms, step increment *E*_step_ 1 mV, step length *t*_step_ 20 ms, and potential scanning window 0–0.5 V). The obtained DPV curve was baseline corrected using a polynomial detrending algorithm.^73^ All data were processed in MATLAB (MATLAB 2019b, MathWorks, USA).

### Electrochemical Testing

Electrochemical stability under pulsed stimulation was evaluated by applying 50,000 biphasic pulses of 10 ms duration each between ±2 V versus Ag/AgCl reference electrode and measuring the impedance before and after the stimulation. Measurements were performed in PBS and in a three-electrode configuration.

### Mechanical Testing

Tensile tests were performed by using a universal testing machine (TesT, Germany) to evaluate the mechanical robustness of the sensors. The sensors were bent at a constant speed of 10 mm min^–1^ for 100 consecutive cycles, and electrochemical impedance was measured before and after this cyclic compression test.

## Supporting Information Available

Additional experimental findings, including photos and experimental results on the optimization of laser parameters for LIG formation; UV–vis absorption spectrum of pristine ZIF-67; Raman spectra of PI_L_, PI+Z67_L_, Z67_pristine_, and Z67_O_ under different conditions; XRD analysis of Z67_O_, PI+Z67_L_, and PI+Z67_L_ as prepared, as washed, and after use; BET (Krypton adsorption) analysis of laser-scribed PI_L_; XPS analysis of the Cl 2p and F 1s core levels; SEM images of PI_L_ and Z67_pristine_; HAADF-STEM images of PI+Z67_L_ and Z67_O_ before and after use; TEM images of Z67_O_; thickness profiles; CV and DPV of sensors at different Z67 loadings; and EIS after stimulation pulses (PDF)

## References

[ref1] Venton B. J.; Wightman R. M. Psychoanalytical Electrochemistry: Dopamine and Behavior. Anal. Chem. 2003, 75 (19), 414A–421A. 10.1021/ac031421c.

[ref2] Gao W.; Emaminejad S.; Nyein H. Y. Y.; Challa S.; Chen K.; Peck A.; Fahad H. M.; Ota H.; Shiraki H.; Kiriya D.; Lien D.-H.; Brooks G. A.; Davis R. W.; Javey A. Fully Integrated Wearable Sensor Arrays for Multiplexed in Situ Perspiration Analysis. Nature 2016, 529 (7587), 509–514. 10.1038/nature16521. 26819044 PMC4996079

[ref3] Yew Y. T.; Ambrosi A.; Pumera M. Nitroaromatic Explosives Detection Using Electrochemically Exfoliated Graphene. Sci. Rep 2016, 6, 33276 10.1038/srep33276. 27633489 PMC5025880

[ref4] Pumera M. Electrochemistry of Graphene, Graphene Oxide and Other Graphenoids: Review. Electrochem. Commun. 2013, 36, 14–18. 10.1016/j.elecom.2013.08.028.

[ref5] Rahmati M.; Mozafari M. Biological Response to Carbon-Family Nanomaterials: Interactions at the Nano-Bio Interface. Front Bioeng Biotechnol 2019, 7, 4 10.3389/fbioe.2019.00004. 30729107 PMC6351449

[ref6] Krejci J.; Sajdlova Z.; Nedela V.; Flodrova E.; Sejnohova R.; Vranova H.; Plicka R. Effective Surface Area of Electrochemical Sensors. J. Electrochem. Soc. 2014, 161 (6), B147 10.1149/2.091406jes.

[ref7] Bakker E.; Telting-Diaz M. Electrochemical Sensors. Anal. Chem. 2002, 74 (12), 2781–2800. 10.1021/ac0202278. 12090665

[ref8] Abdel-Aziz A. M.; Hassan H. H.; Badr I. H. A. Activated Glassy Carbon Electrode as an Electrochemical Sensing Platform for the Determination of 4-Nitrophenol and Dopamine in Real Samples. ACS Omega 2022, 7 (38), 34127–34135. 10.1021/acsomega.2c03427. 36188318 PMC9520556

[ref9] Cui F.; Zhang X. Electrochemical Sensor for Epinephrine Based on a Glassy Carbon Electrode Modified with Graphene/Gold Nanocomposites. J. Electroanal. Chem. 2012, 669, 35–41. 10.1016/j.jelechem.2012.01.021.

[ref10] Gao C.; Guo Z.; Liu J.-H.; Huang X.-J. The New Age of Carbon Nanotubes: An Updated Review of Functionalized Carbon Nanotubes in Electrochemical Sensors. Nanoscale 2012, 4 (6), 1948–1963. 10.1039/c2nr11757f. 22337209

[ref11] Ahammad A. J. S.; Lee J.-J.; Rahman M. A. Electrochemical Sensors Based on Carbon Nanotubes. Sensors 2009, 9 (4), 2289–2319. 10.3390/s90402289. 22574013 PMC3348810

[ref12] Ameku W. A.; Negahdary M.; Lima I. S.; Santos B. G.; Oliveira T. G.; Paixão T. R. L. C.; Angnes L. Laser-Scribed Graphene-Based Electrochemical Sensors: A Review. Chemosensors 2022, 10 (12), 505 10.3390/chemosensors10120505.

[ref13] Behrent A.; Griesche C.; Sippel P.; Baeumner A. J. Process-Property Correlations in Laser-Induced Graphene Electrodes for Electrochemical Sensing. Microchim Acta 2021, 188 (5), 159 10.1007/s00604-021-04792-3. PMC8026455 33829346

[ref14] Lin J.; Peng Z.; Liu Y.; Ruiz-Zepeda F.; Ye R.; Samuel E. L. G.; Yacaman M. J.; Yakobson B. I.; Tour J. M. Laser-Induced Porous Graphene Films from Commercial Polymers. Nat. Commun. 2014, 5 (1), 5714 10.1038/ncomms6714. 25493446 PMC4264682

[ref15] Ye R.; Chyan Y.; Zhang J.; Li Y.; Han X.; Kittrell C.; Tour J. M. Laser-Induced Graphene Formation on Wood. Adv. Mater. 2017, 29 (37), 1702211 10.1002/adma.201702211. 28737226

[ref16] Chyan Y.; Ye R.; Li Y.; Singh S. P.; Arnusch C. J.; Tour J. M. Laser-Induced Graphene by Multiple Lasing: Toward Electronics on Cloth, Paper, and Food. ACS Nano 2018, 12 (3), 2176–2183. 10.1021/acsnano.7b08539. 29436816

[ref17] de la Roche J.; López-Cifuentes I.; Jaramillo-Botero A. Influence of Lasing Parameters on the Morphology and Electrical Resistance of Polyimide-Based Laser-Induced Graphene (LIG). Carbon Lett. 2023, 33 (2), 587–595. 10.1007/s42823-022-00447-2.

[ref18] Houeix Y.; Romero F. J.; Moraila C. L.; Rivadeneyra A.; Rodriguez N.; Morales D. P.; Salinas-Castillo A. Laser-Synthesis of Conductive Carbon-Based Materials from Two Flexible Commercial Substrates: A Comparison. Appl. Surf. Sci. 2023, 634, 157629 10.1016/j.apsusc.2023.157629.

[ref19] Nayak P.; Kurra N.; Xia C.; Alshareef H. N. Highly Efficient Laser Scribed Graphene Electrodes for On-Chip Electrochemical Sensing Applications. Advanced Electronic Materials 2016, 2 (10), 1600185 10.1002/aelm.201600185.

[ref20] Li Y.; Luong D. X.; Zhang J.; Tarkunde Y. R.; Kittrell C.; Sargunaraj F.; Ji Y.; Arnusch C. J.; Tour J. M. Laser-Induced Graphene in Controlled Atmospheres: From Superhydrophilic to Superhydrophobic Surfaces. Adv. Mater. 2017, 29 (27), 1700496 10.1002/adma.201700496. 28497883

[ref21] Li L.; Zhang J.; Peng Z.; Li Y.; Gao C.; Ji Y.; Ye R.; Kim N. D.; Zhong Q.; Yang Y.; Fei H.; Ruan G.; Tour J. M. High-Performance Pseudocapacitive Microsupercapacitors from Laser-Induced Graphene. Adv. Mater. 2016, 28 (5), 838–845. 10.1002/adma.201503333. 26632264

[ref22] Zhu J.; Guo X.; Wang H.; Song W. Cost-Effective Fabrication and High-Frequency Response of Non-Ideal RC Application Based on 3D Porous Laser-Induced Graphene. J. Mater. Sci. 2018, 53 (17), 12413–12420. 10.1007/s10853-018-2514-y.

[ref23] Luong D. X.; Subramanian A. K.; Silva G. A. L.; Yoon J.; Cofer S.; Yang K.; Owuor P. S.; Wang T.; Wang Z.; Lou J.; Ajayan P. M.; Tour J. M. Laminated Object Manufacturing of 3D-Printed Laser-Induced Graphene Foams. Adv. Mater. 2018, 30 (28), 1707416 10.1002/adma.201707416. 29845669

[ref24] Luo J.; Yao Y.; Duan X.; Liu T. Force and Humidity Dual Sensors Fabricated by Laser Writing on Polyimide/Paper Bilayer Structure for Pulse and Respiration Monitoring. J. Mater. Chem. C 2018, 6 (17), 4727–4736. 10.1039/C8TC00457A.

[ref25] Movaghgharnezhad S.; Kang P. Laser-Induced Graphene: Synthesis Advances, Structural Tailoring, Enhanced Properties, and Sensing Applications. J. Mater. Chem. C 2024, 12 (19), 6718–6742. 10.1039/D3TC04677J.

[ref26] Ulhakim M. T.; Rezki M.; Dewi K. K.; Abrori S. A.; Harimurti S.; Septiani N. L. W.; Kurnia K. A.; Setyaningsih W.; Darmawan N.; Yuliarto B. Review—Recent Trend on Two-Dimensional Metal-Organic Frameworks for Electrochemical Biosensor Application. J. Electrochem. Soc. 2020, 167 (13), 136509 10.1149/1945-7111/abb6cc.

[ref27] Chang J.; Wang X.; Wang J.; Li H.; Li F. Nucleic Acid-Functionalized Metal–Organic Framework-Based Homogeneous Electrochemical Biosensor for Simultaneous Detection of Multiple Tumor Biomarkers. Anal. Chem. 2019, 91 (5), 3604–3610. 10.1021/acs.analchem.8b05599. 30757896

[ref28] Liu T.-Z.; Hu R.; Zhang X.; Zhang K.-L.; Liu Y.; Zhang X.-B.; Bai R.-Y.; Li D.; Yang Y.-H. Metal–Organic Framework Nanomaterials as Novel Signal Probes for Electron Transfer Mediated Ultrasensitive Electrochemical Immunoassay. Anal. Chem. 2016, 88 (24), 12516–12523. 10.1021/acs.analchem.6b04191. 28193012

[ref29] Asaduzzaman M.; Faruk O.; Samad A. A.; Kim H.; Reza M. S.; Lee Y.; Park J. Y. A MOFs-Derived Hydroxyl-Functionalized Hybrid Nanoporous Carbon Incorporated Laser-Scribed Graphene-Based Multimodal Skin Patch for Perspiration Analysis and Electrocardiogram Monitoring. Adv. Funct. Mater. 2024, 34 (40), 2405651 10.1002/adfm.202405651.

[ref30] Guo S.; Gao M.; Zhang W.; Liu F.; Guo X.; Zhou K. Recent Advances in Laser-Induced Synthesis of MOF Derivatives. Adv. Mater. 2023, 35 (52), 2303065 10.1002/adma.202303065. 37319033

[ref31] Arthanari S.; Sivaprakasam R.; Park J.-E.; Yang M.; Lee H.; Kim B.-S.; Hwang J. S. Fabrication of Porous Non-Enzymatic Glucose Sensing Electrodes Through Nanosecond-Laser Patterning of Metal–Organic Frameworks. Advanced Materials Technologies 2024, 9 (5), 2301561 10.1002/admt.202301561.

[ref32] Guo S.; Zhao Y.; Yuan H.; Wang C.; Jiang H.; Cheng G. J. Ultrafast Laser Manufacture of Stable, Efficient Ultrafine Noble Metal Catalysts Mediated with MOF Derived High Density Defective Metal Oxides. Small 2020, 16 (18), 2000749 10.1002/smll.202000749. 32285619

[ref33] Lam D. V.; Nguyen U. N. T.; Roh E.; Choi W.; Kim J.-H.; Kim H.; Lee S.-M. Graphitic Carbon with MnO/Mn7C3 Prepared by Laser-Scribing of MOF for Versatile Supercapacitor Electrodes. Small 2021, 17 (29), 2100670 10.1002/smll.202100670. 34145746

[ref34] Lam D. V.; Sohail M.; Nguyen V.-T.; Ngo Q.-T.; Anto Jeffery A.; Choi H.-S.; Jung N.; Kim J.-H.; Kim H.; Lee S.-M. Laser-Scribed Ultrasmall Nanoparticles with Unary and Binary Phases. Chemical Engineering Journal 2021, 421, 127731 10.1016/j.cej.2020.127731.

[ref35] Van Lam D.; Sohail M.; Kim J.-H.; Lee H. J.; Han S. O.; Shin J.; Kim D.; Kim H.; Lee S.-M. Laser Synthesis of MOF-Derived Ni@Carbon for High-Performance Pseudocapacitors. ACS Appl. Mater. Interfaces 2020, 12 (35), 39154–39162. 10.1021/acsami.0c10235. 32805916

[ref36] Zhang W.; Li R.; Zheng H.; Bao J.; Tang Y.; Zhou K. Laser-Assisted Printing of Electrodes Using Metal–Organic Frameworks for Micro-Supercapacitors. Adv. Funct. Mater. 2021, 31, 2009057 10.1002/adfm.202009057.

[ref37] Wu Y.; Huang Z.; Jiang H.; Wang C.; Zhou Y.; Shen W.; Xu H.; Deng H. Facile Synthesis of Uniform Metal Carbide Nanoparticles from Metal–Organic Frameworks by Laser Metallurgy. ACS Appl. Mater. Interfaces 2019, 11 (47), 44573–44581. 10.1021/acsami.9b13864. 31661951

[ref38] Tang Y.-J.; Zheng H.; Wang Y.; Zhang W.; Zhou K. Laser-Induced Annealing of Metal–Organic Frameworks on Conductive Substrates for Electrochemical Water Splitting. Adv. Funct. Mater. 2021, 31 (31), 2102648 10.1002/adfm.202102648.

[ref39] Gao S.; Ji W.; Zhu Q.; Jarlöv A.; Shen X.; Bai X.; Zhu C.; Lek Y. Z.; Xiao Z.; Zhou K. Pulsed-Wave Laser Additive Manufacturing of CrCoNi Medium-Entropy Alloys with High Strength and Ductility. Mater. Today 2024, 81, 36 10.1016/j.mattod.2024.10.004.

[ref40] Guo Y.; Zhang C.; Chen Y.; Nie Z. Research Progress on the Preparation and Applications of Laser-Induced Graphene Technology. Nanomaterials (Basel) 2022, 12 (14), 2336 10.3390/nano12142336. 35889560 PMC9317010

[ref41] Stanford M. G.; Yang K.; Chyan Y.; Kittrell C.; Tour J. M. Laser-Induced Graphene for Flexible and Embeddable Gas Sensors. ACS Nano 2019, 13 (3), 3474–3482. 10.1021/acsnano.8b09622. 30848881

[ref42] Ye R.; James D. K.; Tour J. M. Laser-Induced Graphene. Acc. Chem. Res. 2018, 51 (7), 1609–1620. 10.1021/acs.accounts.8b00084. 29924584

[ref43] Ye R.; James D. K.; Tour J. M. Laser-Induced Graphene: From Discovery to Translation. Adv. Mater. 2019, 31 (1), 1803621 10.1002/adma.201803621. 30368919

[ref44] Carvalho A. F.; Fernandes A. J. S.; Leitão C.; Deuermeier J.; Marques A. C.; Martins R.; Fortunato E.; Costa F. M. Laser-Induced Graphene Strain Sensors Produced by Ultraviolet Irradiation of Polyimide. Adv. Funct. Mater. 2018, 28 (52), 1805271 10.1002/adfm.201805271.

[ref45] Santos N. F.; Pereira S. O.; Moreira A.; Girão A. V.; Carvalho A. F.; Fernandes A. J. S.; Costa F. M. IR and UV Laser-Induced Graphene: Application as Dopamine Electrochemical Sensors. Advanced Materials Technologies 2021, 6 (6), 2100007 10.1002/admt.202100007.

[ref46] Liu X.; Yin B.; Yang C.; Wu S. Passivation Strategies for Enhancing Sensitivity and Repeatability of Microelectrode Electrochemical Sensors. Talanta 2024, 273, 125946 10.1016/j.talanta.2024.125946. 38508127

[ref47] Jiang H.; Jin S.; Wang C.; Ma R.; Song Y.; Gao M.; Liu X.; Shen A.; Cheng G. J.; Deng H. Nanoscale Laser Metallurgy and Patterning in Air Using MOFs. J. Am. Chem. Soc. 2019, 141 (13), 5481–5489. 10.1021/jacs.9b00355. 30823704

[ref48] Thapliyal V.; Alabdulkarim M. E.; Whelan D. R.; Mainali B.; Maxwell J. L. A Concise Review of the Raman Spectra of Carbon Allotropes. Diamond Relat. Mater. 2022, 127, 109180 10.1016/j.diamond.2022.109180.

[ref49] Ferrari A. C.; Robertson J. Interpretation of Raman Spectra of Disordered and Amorphous Carbon. Phys. Rev. B 2000, 61 (20), 14095–14107. 10.1103/PhysRevB.61.14095.

[ref50] Kong L.; Chen Q.; Shen X.; Xu Z.; Xu C.; Ji Z.; Zhu J. MOF Derived Nitrogen-Doped Carbon Polyhedrons Decorated on Graphitic Carbon Nitride Sheets with Enhanced Electrochemical Capacitive Energy Storage Performance. Electrochim. Acta 2018, 265, 651–661. 10.1016/j.electacta.2018.01.146.

[ref51] Maulana A. Y.; Kim S.; Shim J.-H.; Lee C.; Song J.; Lee D.-W.; Yun B.; Gim H.; Futalan C. M.; Kim J. N-Doped Graphitic Carbon Encapsulated Cobalt Oxide Nanoparticles from ZIF-67 on ZIF-8 as an Anode Material for Li-Ion Batteries. J. Alloys Compd. 2022, 908, 164645 10.1016/j.jallcom.2022.164645.

[ref52] Hussain M. Z.; Pawar G. S.; Huang Z.; Tahir A. A.; Fischer R. A.; Zhu Y.; Xia Y. Porous ZnO/Carbon Nanocomposites Derived from Metal Organic Frameworks for Highly Efficient Photocatalytic Applications: A Correlational Study. Carbon 2019, 146, 348–363. 10.1016/j.carbon.2019.02.013.

[ref53] Aijaz A.; Masa J.; Rösler C.; Xia W.; Weide P.; Botz A. J. R.; Fischer R. A.; Schuhmann W.; Muhler M. Co@Co3O4 Encapsulated in Carbon Nanotube-Grafted Nitrogen-Doped Carbon Polyhedra as an Advanced Bifunctional Oxygen Electrode. Angew. Chem., Int. Ed. 2016, 55 (12), 4087–4091. 10.1002/anie.201509382. 26913583

[ref54] Das R.; Pachfule P.; Banerjee R.; Poddar P. Metal and Metal Oxide Nanoparticle Synthesis from Metal Organic Frameworks (MOFs): Finding the Border of Metal and Metal Oxides. Nanoscale 2012, 4 (2), 591–599. 10.1039/C1NR10944H. 22143166

[ref55] Hussain M. Z.; Yang Z.; Huang Z.; Jia Q.; Zhu Y.; Xia Y. Recent Advances in Metal–Organic Frameworks Derived Nanocomposites for Photocatalytic Applications in Energy and Environment. Advanced Science 2021, 8 (14), 2100625 10.1002/advs.202100625. 34032017 PMC8292888

[ref56] Gunina E. V.; Zhestkij N. A.; Sergeev M.; Bachinin S. V.; Mezenov Y. A.; Kulachenkov N. K.; Timofeeva M.; Ivashchenko V.; Timin A. S.; Shipilovskikh S. A.; Yakubova A. A.; Pavlov D. I.; Potapov A. S.; Gong J.; Khamkhash L.; Atabaev T. Sh.; Bruyere S.; Milichko V. A. Laser-Assisted Design of MOF-Derivative Platforms from Nano- to Centimeter Scales for Photonic and Catalytic Applications. ACS Appl. Mater. Interfaces 2023, 15 (40), 47541–47551. 10.1021/acsami.3c10193. 37773641

[ref57] Records W. C.; Yoon Y.; Ohmura J. F.; Chanut N.; Belcher A. M. Virus-templated Pt–Ni(OH)_2_ nanonetworks for enhanced electrocatalytic reduction of water. Nano Energy 2019, 58, 167–174. 10.1016/j.nanoen.2018.12.083.

[ref58] Biesinger M. C.; Payne B. P.; Grosvenor A. P.; Lau L. W. M.; Gerson A. R.; Smart R. St. C. Resolving Surface Chemical States in XPS Analysis of First Row Transition Metals, Oxides and Hydroxides: Cr, Mn, Fe, Co and Ni. Appl. Surf. Sci. 2011, 257 (7), 2717–2730. 10.1016/j.apsusc.2010.10.051.

[ref59] Tanuma S.; Powell C. J.; Penn D. R. Calculations of Electron Inelastic Mean Free Paths. V. Calculations of Electron Inelastic Mean Free Paths. V.Data for 14 Organic Compounds over the 50–2000 eV Range. Surf. Interface Anal. 1994, 21 (3), 165–176. 10.1002/sia.740210302.

[ref60] Smardz L.; Köbler U.; Zinn W. Oxidation Kinetics of Thin and Ultrathin Cobalt Films. J. Appl. Phys. 1992, 71 (10), 5199–5204. 10.1063/1.351378.

[ref61] Lin Y.; Huang Y.; Chen X. Recent Advances in Metal-Organic Frameworks for Biomacromolecule Sensing. Chemosensors 2022, 10, 412 10.3390/chemosensors10100412.

[ref62] Leiro J. A.; Heinonen M. H.; Laiho T.; Batirev I. G. Core-Level XPS Spectra of Fullerene, Highly Oriented Pyrolitic Graphite, and Glassy Carbon. J. Electron Spectrosc. Relat. Phenom. 2003, 128 (2), 205–213. 10.1016/S0368-2048(02)00284-0.

[ref63] Pels J. R.; Kapteijn F.; Moulijn J. A.; Zhu Q.; Thomas K. M. Evolution of Nitrogen Functionalities in Carbonaceous Materials during Pyrolysis. Carbon 1995, 33 (11), 1641–1653. 10.1016/0008-6223(95)00154-6.

[ref64] Wang W.; Li C.; Zhang G.; Sheng L. Matrix-Assisted Pulsed Laser Evaporation of Polyimide Thin Films and the XPS Study. Sci. China Ser. B-Chem. 2008, 51 (10), 983–989. 10.1007/s11426-008-0011-x.

[ref65] Dang H. T.; Cheong S.; Kim J.; Tran N. T.; Kim H.; Lee H. Tetrachlorocobaltate-Catalyzed Methane Oxidation to Methyl Trifluoroacetate. Catalysts 2022, 12 (11), 1419 10.3390/catal12111419.

[ref66] Lakshmanakumar M.; Nesakumar N.; Kulandaisamy A. J.; Rayappan J. B. B. Principles and Recent Developments in Optical and Electrochemical Sensing of Dopamine: A Comprehensive Review. Measurement 2021, 183, 109873 10.1016/j.measurement.2021.109873.

[ref67] Asif A.; Heiskanen A.; Emnéus J.; Keller S. S. Pyrolytic Carbon Nanograss Electrodes for Electrochemical Detection of Dopamine. Electrochim. Acta 2021, 379, 138122 10.1016/j.electacta.2021.138122.

[ref68] Peltola E.; Heikkinen J.; Sovanto K.; Sainio S.; Aarva A.; Franssila S.; Jokinen V.; Laurila T. SU-8 Based Pyrolytic Carbon for the Electrochemical Detection of Dopamine. J. Mater. Chem. B 2017, 5. 9033 10.1039/C7TB02469J. 32264131

[ref69] Choi H. K.; Choi J.-H.; Yoon J. An Updated Review on Electrochemical Nanobiosensors for Neurotransmitter Detection. Biosensors 2023, 13 (9), 892 10.3390/bios13090892. 37754127 PMC10526534

[ref70] Schindler S.; Bechtold T. Mechanistic Insights into the Electrochemical Oxidation of Dopamine by Cyclic Voltammetry. J. Electroanal. Chem. 2019, 836, 94–101. 10.1016/j.jelechem.2019.01.069.

[ref71] Huang Q.; Dai X.; Guo S.; Ma S.; Zhao C.; Su W. Flexible, Wearable and Sensitive Laser-Induced Graphene Sensors for Human Health Monitoring. Polym. Adv. Technol. 2024, 35 (6), e6473 10.1002/pat.6473.

[ref72] Huang Z.; Yang Z.; Hussain M. Z.; Chen B.; Jia Q.; Zhu Y.; Xia Y. Polyoxometallates@zeolitic-Imidazolate-Framework Derived Bimetallic Tungsten-Cobalt Sulfide/Porous Carbon Nanocomposites as Efficient Bifunctional Electrocatalysts for Hydrogen and Oxygen Evolution. Electrochim. Acta 2020, 330, 135335 10.1016/j.electacta.2019.135335.

[ref73] Górski Ł.; Ciepiela F.; Jakubowska M. Automatic Baseline Correction in Voltammetry. Electrochim. Acta 2014, 136, 195–203. 10.1016/j.electacta.2014.05.076.

