# Correction to “Laser-Induced Metal–Organic Framework-Derived Flexible Electrodes for Electrochemical Sensing”

**DOI:** 10.1021/acsami.5c01326

**Published:** 2025-01-29

**Authors:** Beatrice De Chiara, Fulvia Del Duca, Mian Zahid Hussain, Tim Kratky, Pritam Banerjee, Sarah V. Dummert, Ali Khoshouei, Nicolas Chanut, Hu Peng, George Al Boustani, Lukas Hiendlmeier, Joerg Jinschek, Rob Ameloot, Hendrik Dietz, Bernhard Wolfrum

In our original article, a typographical error was overlooked during the proofreading process. In the Electrochemical Measurements and Analysis section on page 3778, in the electrochemical oxidation of dopamine, the 2e^–^ should have been on the right-hand side of the reaction equation. In the published version, however, it appears on the left-hand side.

Also, the Editorial Office modified the article title to “Laser-Induced Metal–Organic Framework-Derived Flexible Electrodes for Electrochemical Sensing”.

The correct equation is as follows:
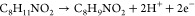


The research findings and conclusions of this work remain unaffected by these corrections.

