# Supplementary material for: Laser-Induced Metal–Organic Framework-Derived Flexible Electrodes for Electrochemical Sensing
Source: ACS Appl Mater Interfaces. 2025 Jan 6;17(2):3772–84. doi: 10.1021/acsami.4c18243 (PMC11744510; doi:10.1021/acsami.4c18243)
Supplement: Supplementary file 1 — am4c18243_si_001.pdf [file am4c18243_si_001.pdf]

Supporting Information

Laser-Induced Metal-Organic Frameworks derived Flexible Electrodes for Electrochemical Sensing

*Beatrice De Chiara[‡1], Fulvia Del Duca[‡1], Mian Zahid Hussain[*2], Tim Kratky[3], Pritam Banerjee[4], Sarah V. Dummert[2], Ali Khoshouei[5], Nicolas Chanut[6], Hu Peng[1], George Al Boustani[1], Lukas Hiendlmeier[1], Joerg Jinschek[4], Rob Ameloot[6], Hendrik Dietz[5], Bernhard Wolfrum[*1]*

[1] Neuroelectronics, Munich Institute of Biomedical Engineering, Department of Electrical Engineering, School of Computation, Information and Technology, Technical University of Munich, Hans-Piloty-Str. 1, 85748 Garching, Germany

[2] Chair of Inorganic and Metal-Organic Chemistry, Department of Chemistry, School of Natural Sciences, Technical University of Munich, Lichtenbergstr. 4, 85748 Garching, Germany

[3] Physical Chemistry with Focus on Catalysis, Department of Chemistry, School of Natural Sciences, Technical University of Munich, Lichtenbergstr 4, Garching, 85748, Germany

[4] National Centre for Nano Fabrication and Characterization (DTU Nanolab), Technical University of Denmark, Fysikvej 307, DK-2800 Kongens Lyngby, Denmark

[5] Laboratory for Biomolecular Nanotechnology, Department of Biosciences, School of Natural Sciences, Technical University of Munich, Am Coulombwall 4a, 85748 Garching, Germany

[6] Center for Membrane Separations, Adsorption, Catalysis and Spectroscopy (cMACS), KU Leuven, 3001 Leuven, Belgium

Corresponding Authors

*Mian Zahid Hussain (*zahid.hussain@tum.de*)

*Bernhard Wolfrum (*bernhard.wolfrum@tum.de*)

‡These authors contributed equally.

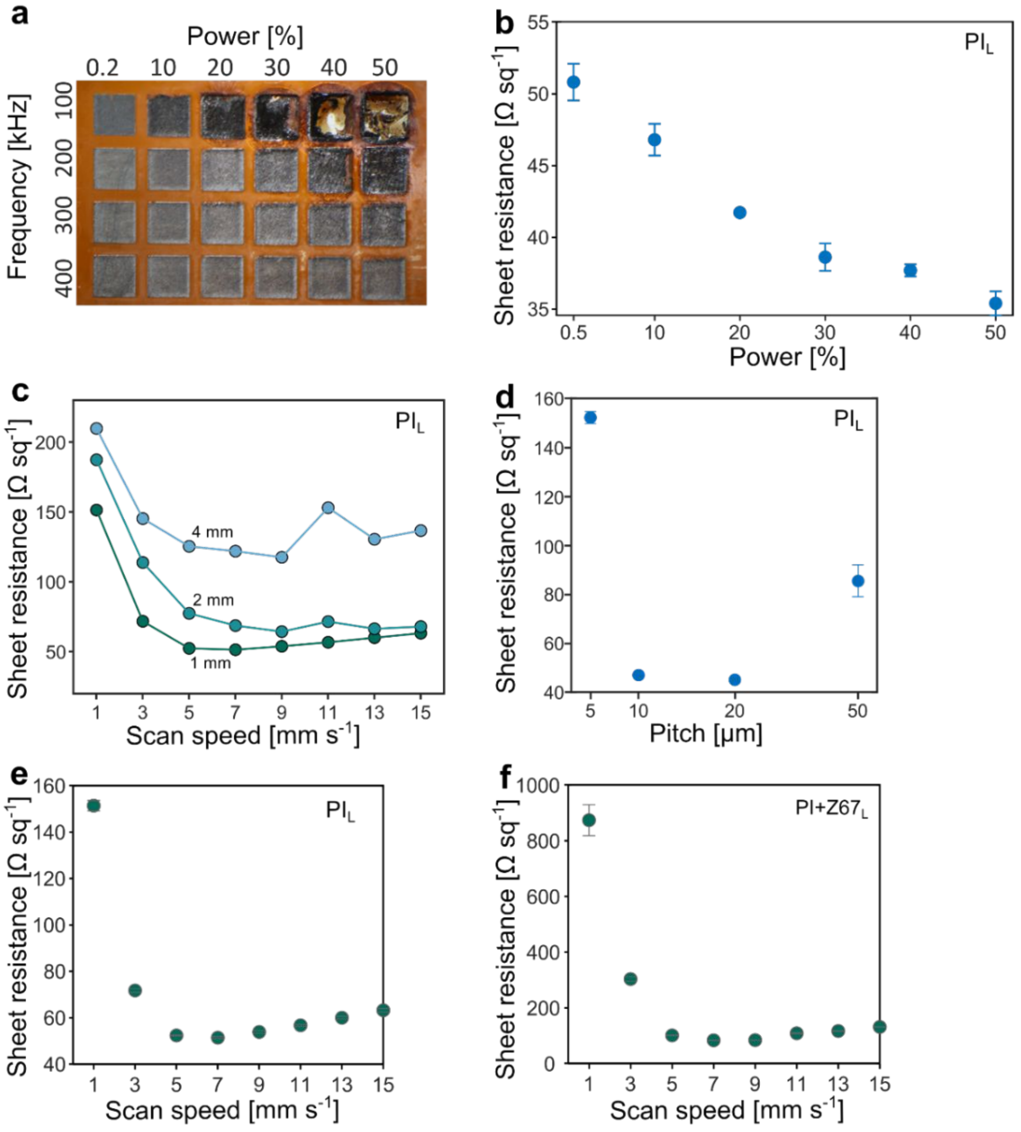

Figure S1. Overview of UV laser parameter optimization for laser scribing process PI and PI+Z67. (a) Real image of the fabricated sample at different laser frequency and power conditions on PI. As shown, the 400 kHz frequency gives better results. The sheet resistance is shown as a function

of (b) laser power, (c) scan speed as a function of different defocus, (d) line pitch and (e) scan speed for the PI. (f) Sheet resistance as a function of scan speed for PI+Z67. The results lead to the selection of the following laser parameters: 10% power, 400 kHz, 1 mm defocus, 20 μm line pitch, and 7 mm/s scan speed. The optimal parameters were chosen to minimize the sheet resistance and to maximize the structural stability.

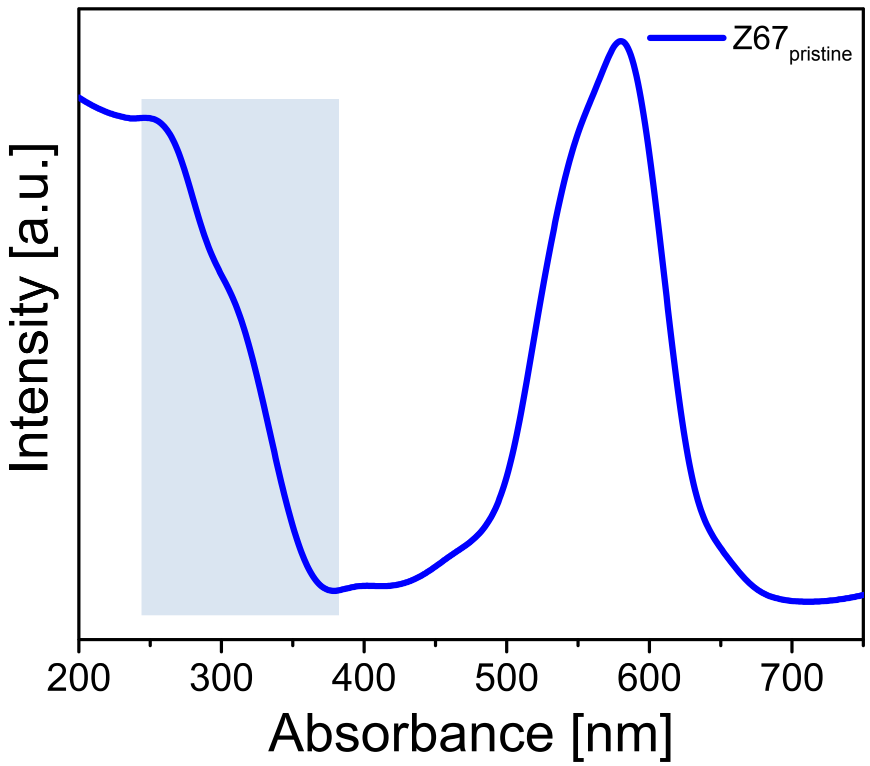

Figure S2. Diffuse reflectance UV-Vis absorption Spectrum of pristine ZIF-67 (powder sample). The absorption between 275-375 nm is due to the ligand-to-metal charge-transfer (LMCT). The second absorption peak represents the d-d ligand field transitions [$^4A_2(F)-^4T_1(P)$] in metal nodes.[1] While the material shows only moderate direct absorption at 355 nm, the actual photothermolysis process is more complex than simple linear absorption. During photothermolysis, UV laser beam delivers a high flux of energetic photons (355 nm) in nanosecond pulses. Even limited initial absorption by the Co-containing nodes and ligand-to-metal charge transfer (LMCT) transitions is sufficient to initiate localized heating. As these excited states relax, electron-electron and electron-phonon interactions rapidly convert the absorbed photon energy into thermal energy, driving bond cleavage of organic linkers and reduction of cobalt centers. This rapid, spatially confined heating

leads ZIF-67 decomposition and transformation into a $Co/Co_3O_4/C$ composite. Thus, even though the starting ZIF-67 may be transparent or only weakly absorbing at 355 nm, the high photon flux, the rapid sequence of absorption-heating-decomposition, and the dynamic changes in local structure collectively ensure effective photothermolysis under the given laser irradiation conditions.

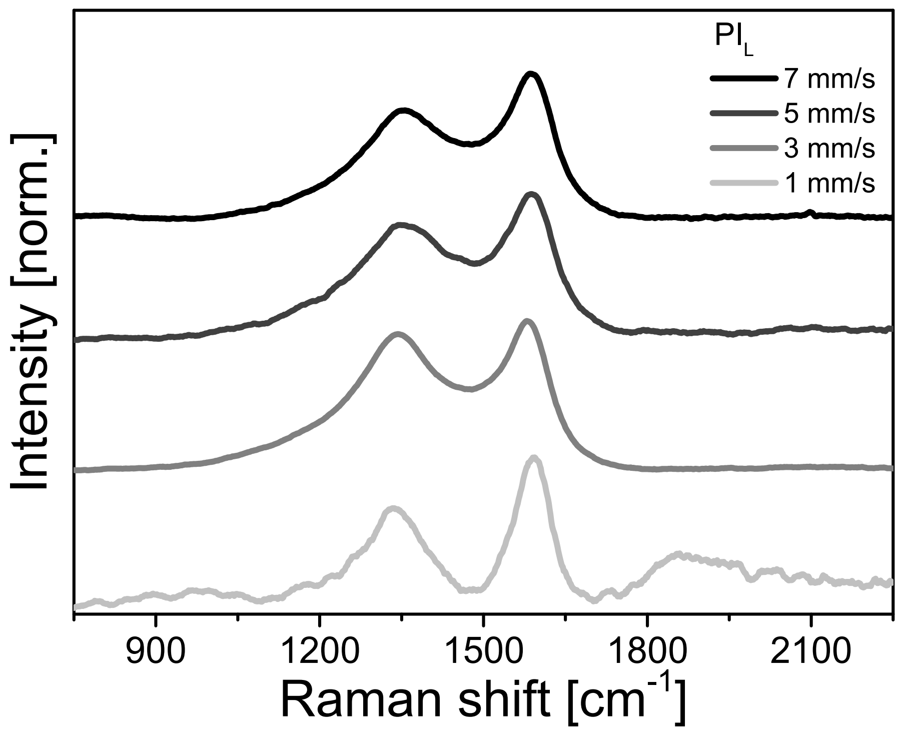

Figure S3. Raman spectra of laser-scribed polyimide substrate (PI$_L$) with different laser speed.

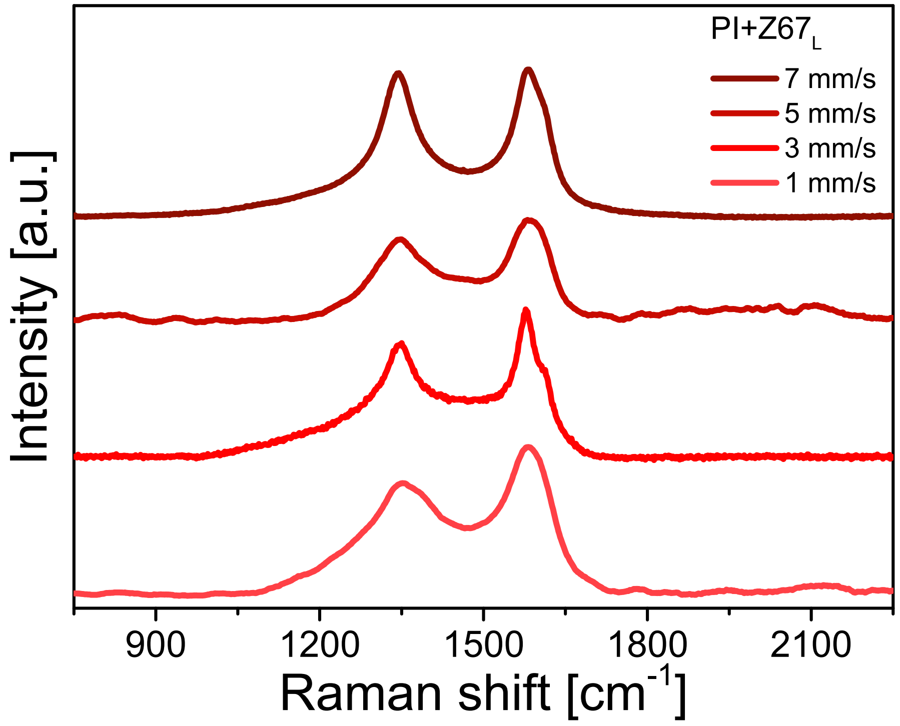

Figure S4. Raman spectra of laser-scribed polyimide substrate coated with ZIF-67 ($PI+Z67_L$) with different laser speed.

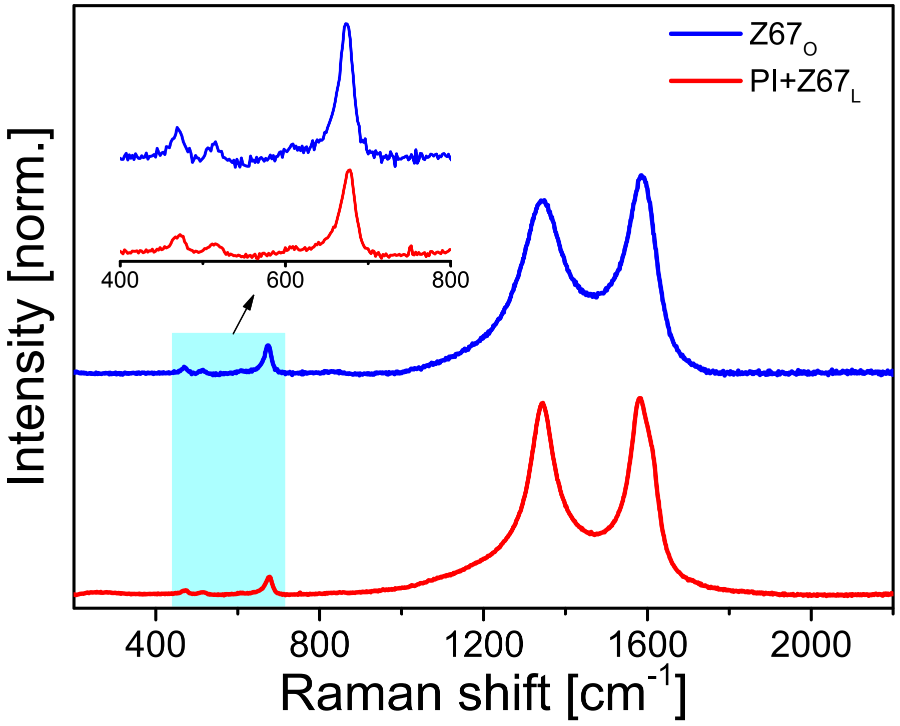

Figure S5. A comparison of Raman spectra of laser-scribed polyimide substrate coated with ZIF-67 ($PI+Z67_L$) at 7 mm/s and oven-pyrolyzed ZIF-67 under an inert atmosphere at 900 ˚C.

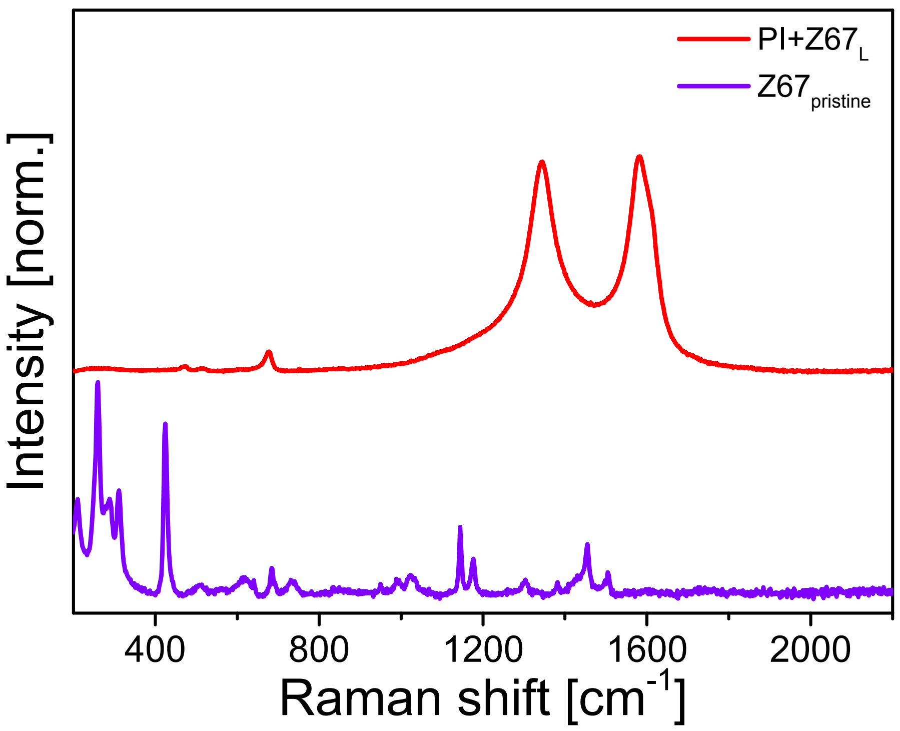

Figure S6. Raman spectra of pristine ZIF-67 and laser-scribed ZIF-67 (PI+Z67$_L$) at 7 mm/s.

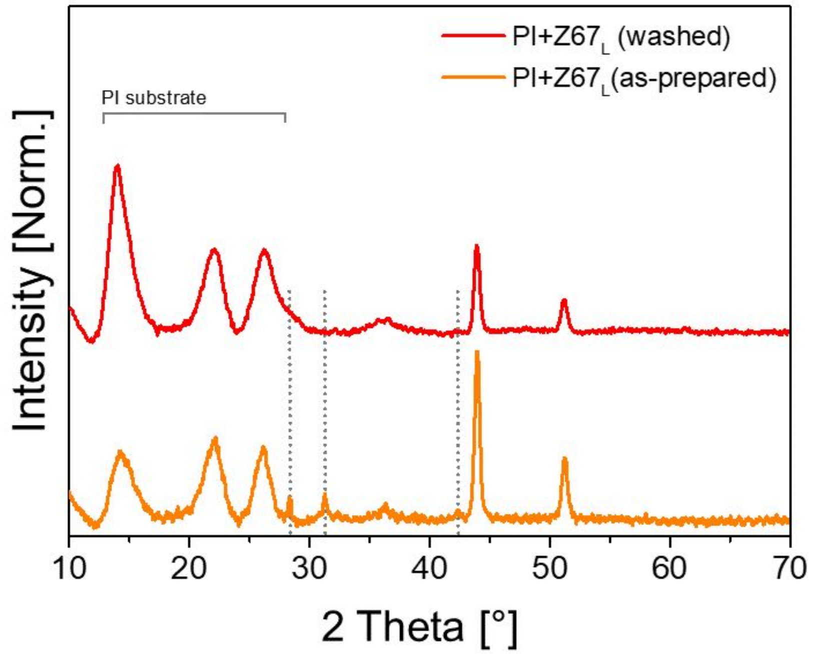

Figure S7. XRD patterns of as-prepared PI+Z67$_L$ and washed samples (with deionized water). It shows that the as-prepared sample contains some traces of $CoCl_2$ and CoF_2. The chlorine (Cl) comes from parylene-C used as a passivation layer. The small amount of fluorine (F) is due to the Nafion being used as a binder to prepare ZIF-67 ink. Since $CoCl_2$ and CoF_2 are sparingly soluble in water, these cobalt halides were easily removed by washing the samples with deionized water. The XRD patterns confirm their successful removal.

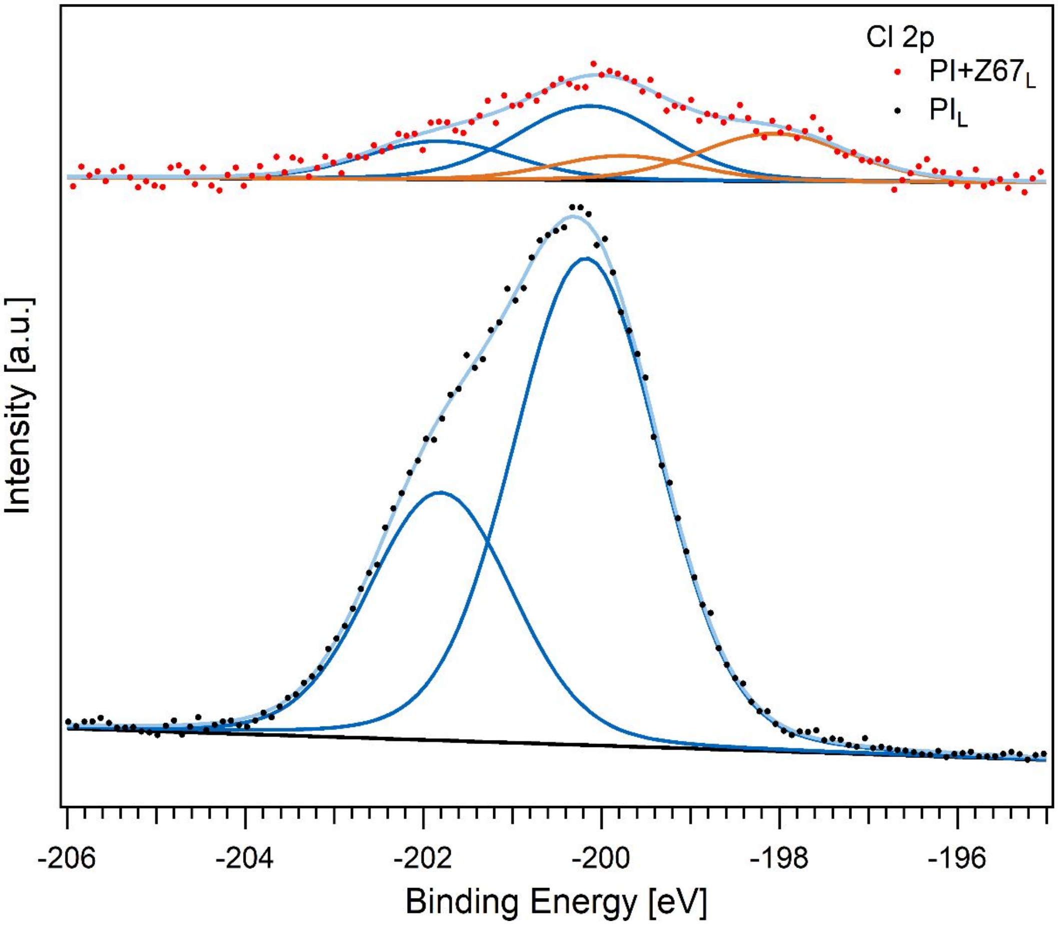

Figure S8. XPS spectra of the Cl 2p core level of laser-scribed PI_L and $PI+Z67_L$ after rinsing in water overnight. The intensity of the spectra is scaled to the total photoemission intensities of the respective C 1s core level. While the blue spin orbit-split components reflect Cl species from parylene-C residues, the orange components appear at substantially lower binding energies characteristic for transition metal chlorides.[2] As $CoCl_2$ was removed upon the rinsing procedure, the remaining features at low binding energy indicate coordination of the Co particles to the support. Hence, the Cl species originating from parylene-C residues might act as an anchoring site for the Co nanoparticles.

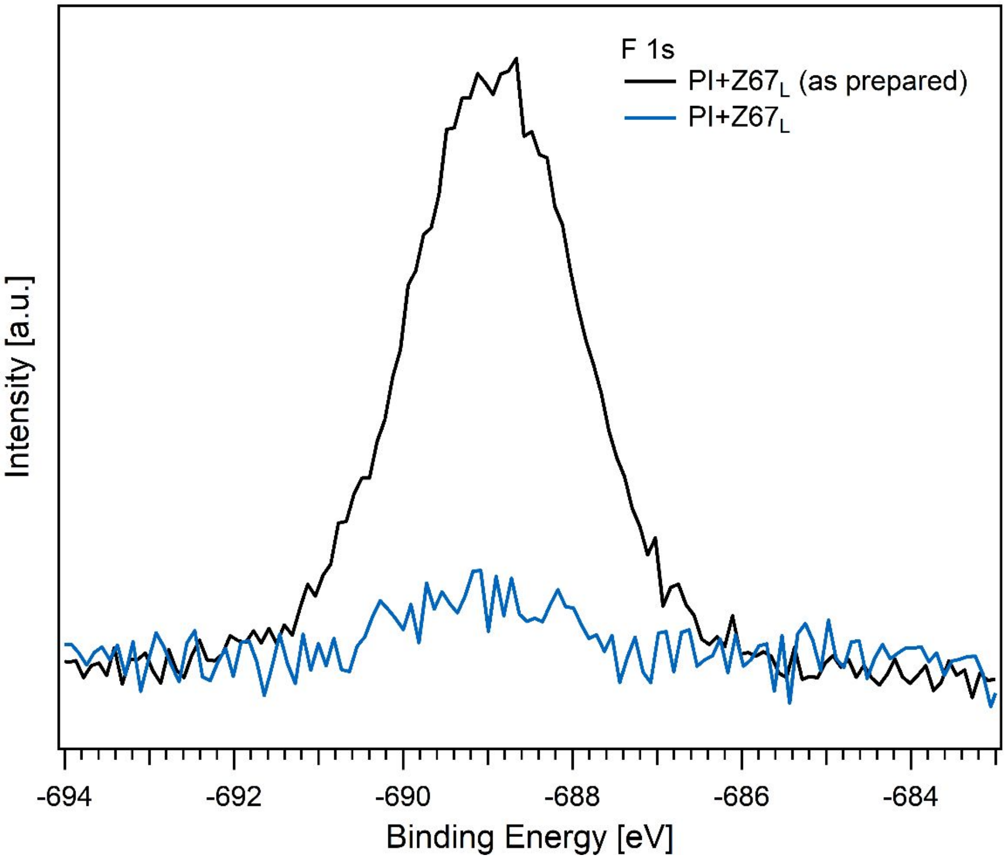

Figure S9. XPS spectra of the F 1s core level of laser-scribed PI+Z67$_L$ and unwashed PI+Z67$_L$ (as-prepared). The intensity of the spectra is scaled to the total photoemission intensities of the respective C 1s core level. The F 1s peaks of both samples appear at a binding energy of ~ 689 eV, which is expected for polytetrafluoroethylene compounds. The photoemission from F 1s is explained by Nafion used as a binder to immobilize the ZIF-67 ink on the PI substrate. The Nafion residues are easily removed by rinsing the sample with water, as observed by the substantial decrease of the F 1s intensity by one order of magnitude.

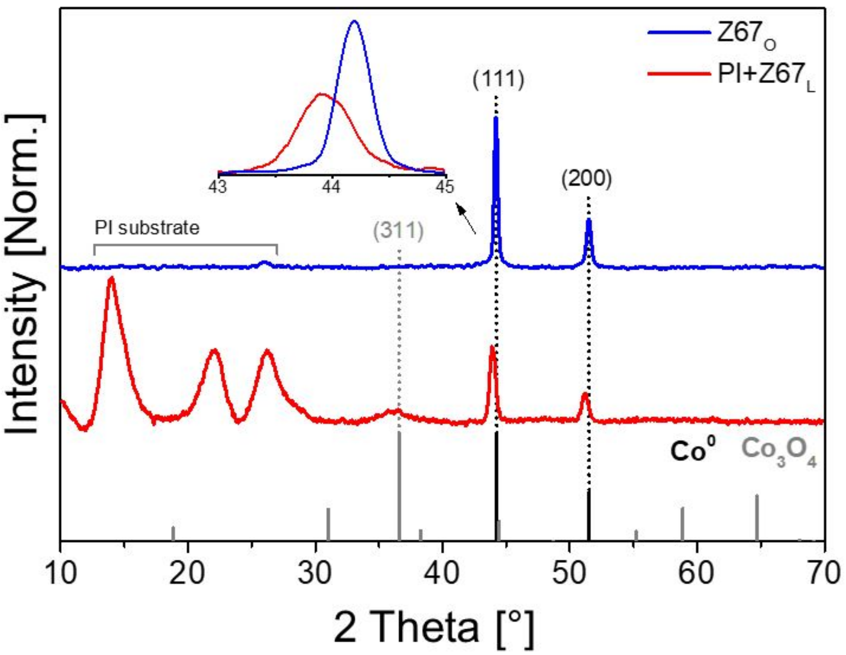

Figure S10. XRD spectra of laser-scribed ZIF-67 coated on polyimide substrate (PI+$Z67_L$) (under ambient) and oven-pyrolyzed ZIF-67 under an inert atmosphere at 900 ˚C.

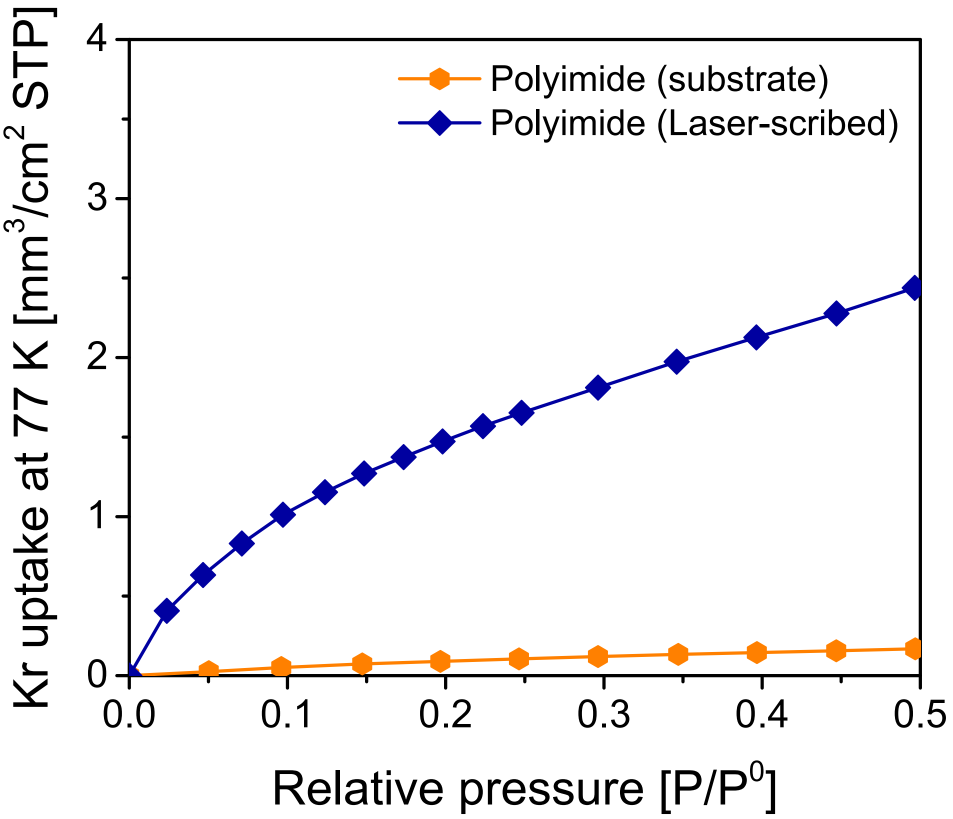

Figure S11. The Krypton adsorption curves of polyimide (PI) substrate and laser-scribed (PI_L).

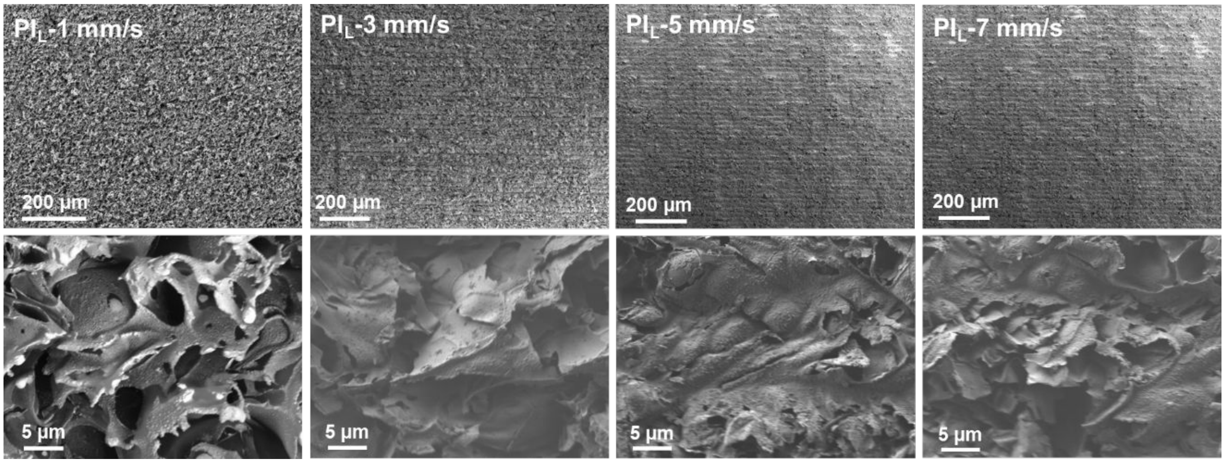

Figure S12. SEM images of laser-scribed polyimide (PI$_L$) with different speeds. The slower speed results in the formation ofgraphitic foam-like structures with a rougher surface.

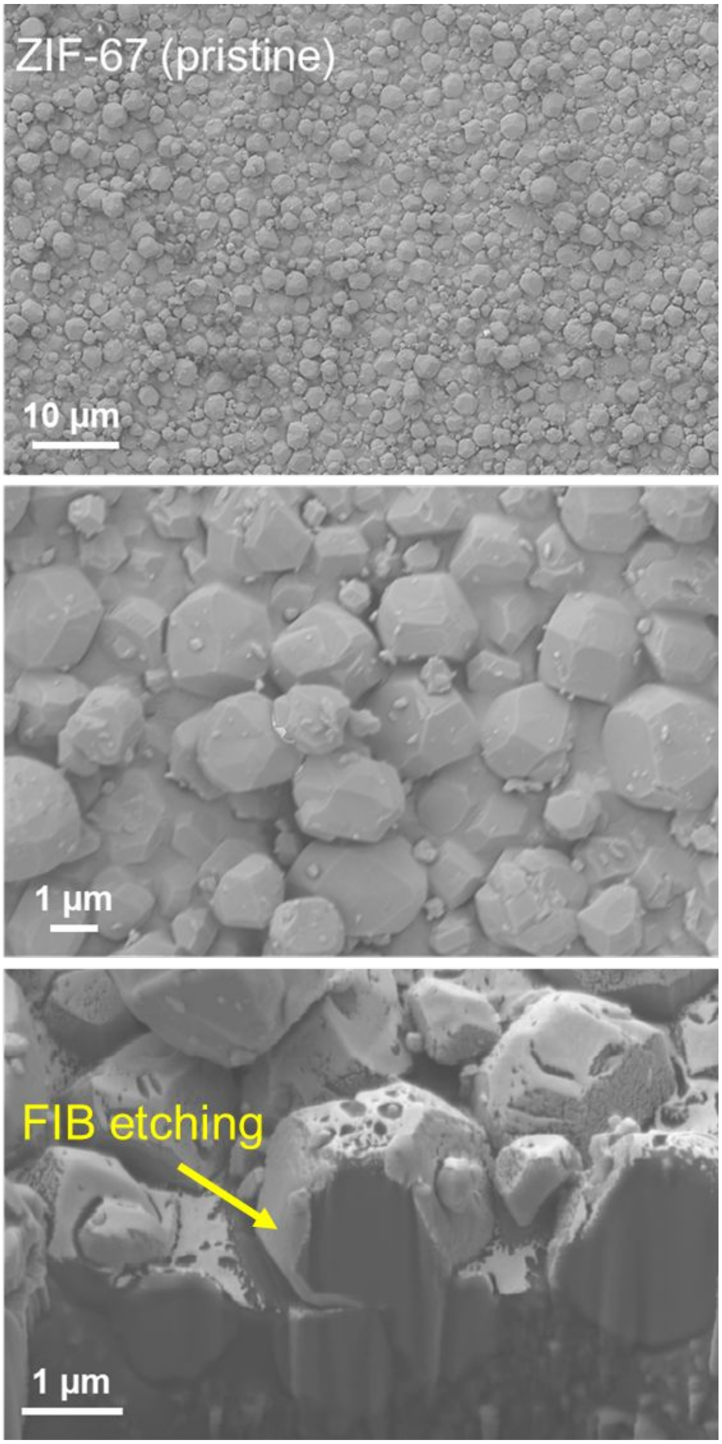

Figure S13. Selected SEM images of pristine ZIF-67 deposited on PI substrate. The FIB-SEM image (bottom) shows well-defined polyhedral morphology of ZIF-67 particles with a solid interior.

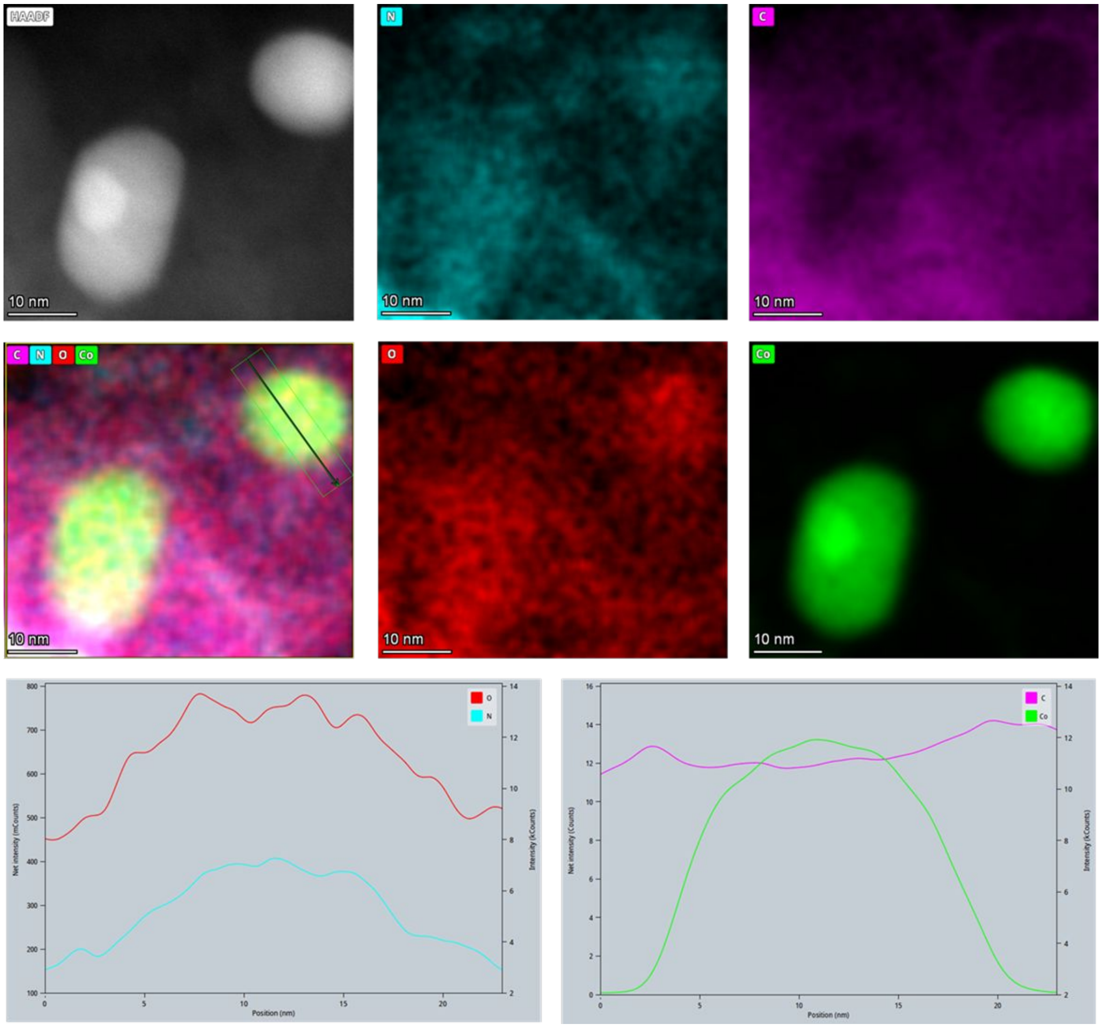

Figure S14 The HAADF STEM-EDX maps and intensity line scan of selected Co NP. The elemental maps of Co, N, O, and C clearly show that the nanoparticle is enriched with Co with a small fraction of O throughout the nanoparticle. The intensity of the Carbon around the nanoparticle is higher. The intensity line profile of the elements in a selected particle also depicts the same behavior. Since the intensity of the O is much lower than the Co, it clearly indicates that a thin oxide layer is present on the surface of Co nanoparticle. Oxygen is also present throughout the nanoparticle because we observe the projection of the nanoparticle in TEM analysis.

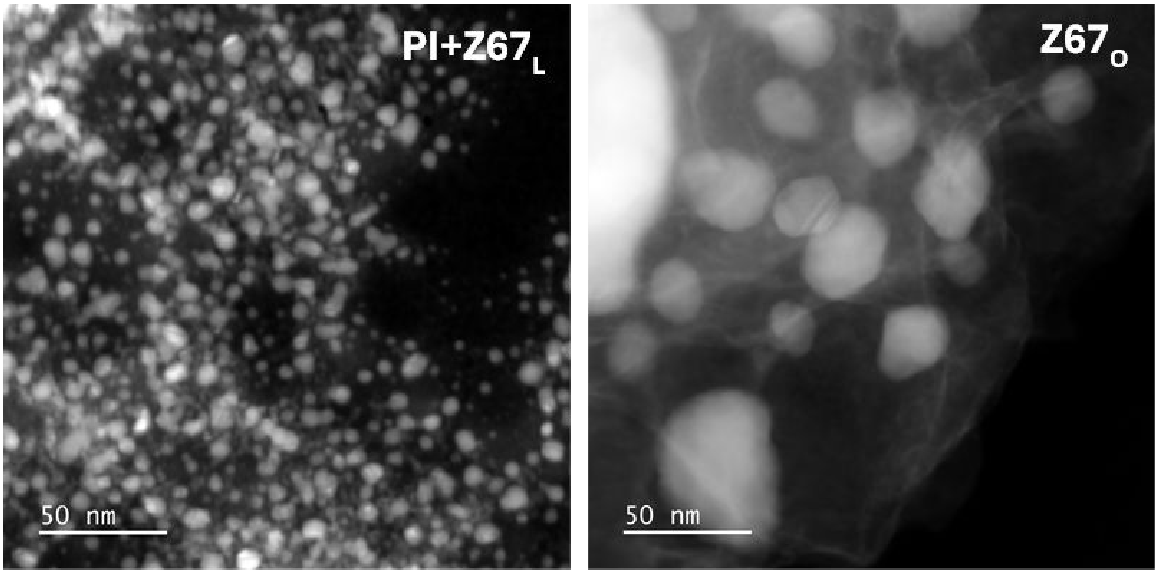

Figure S15. Scanning transmission electron microscopy (HAADF-STEM) images of laser-scribed PI+Z67$_L$ (left) and oven-pyrolyzed Z67$_O$ (right). The particle size of Co/Co_3O_4 in PI+Z67$_L$ is between 5-15 nm (left), whereas in oven-pyrolyzed sample (Z67$_O$) exhibits larger particle size between 20-60 nm due to coalescing effect (right).

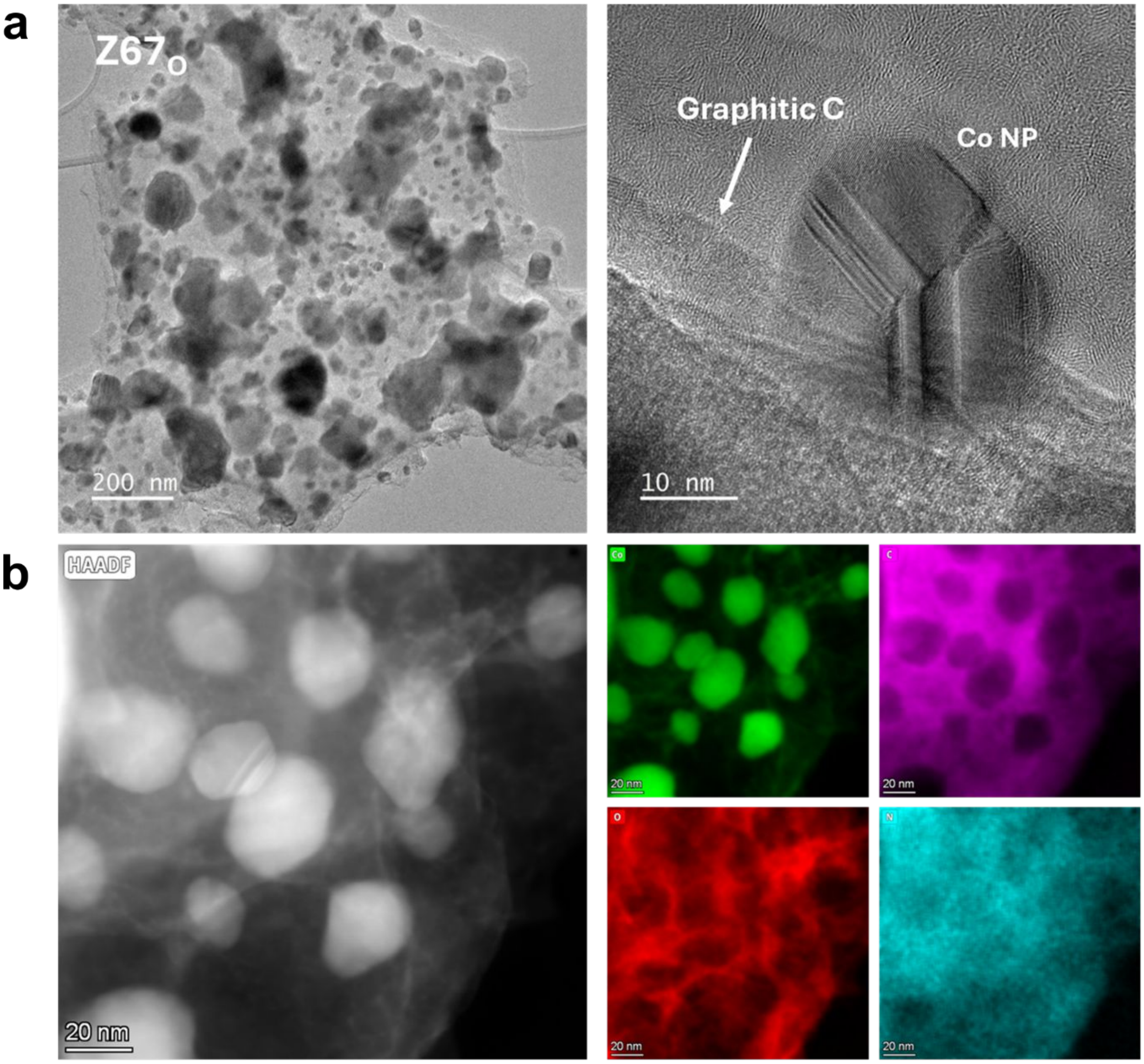

Figure S16. (a) TEM images of oven-pyrolyzed sample $Z67_O$ (left) particle distribution of NPs, (right) Co NP deposited in graphitic carbon (b) HAADF-STEM image (left) and STEM-EDX elemental maps (right) show a uniform distribution of Co, C, O and N.

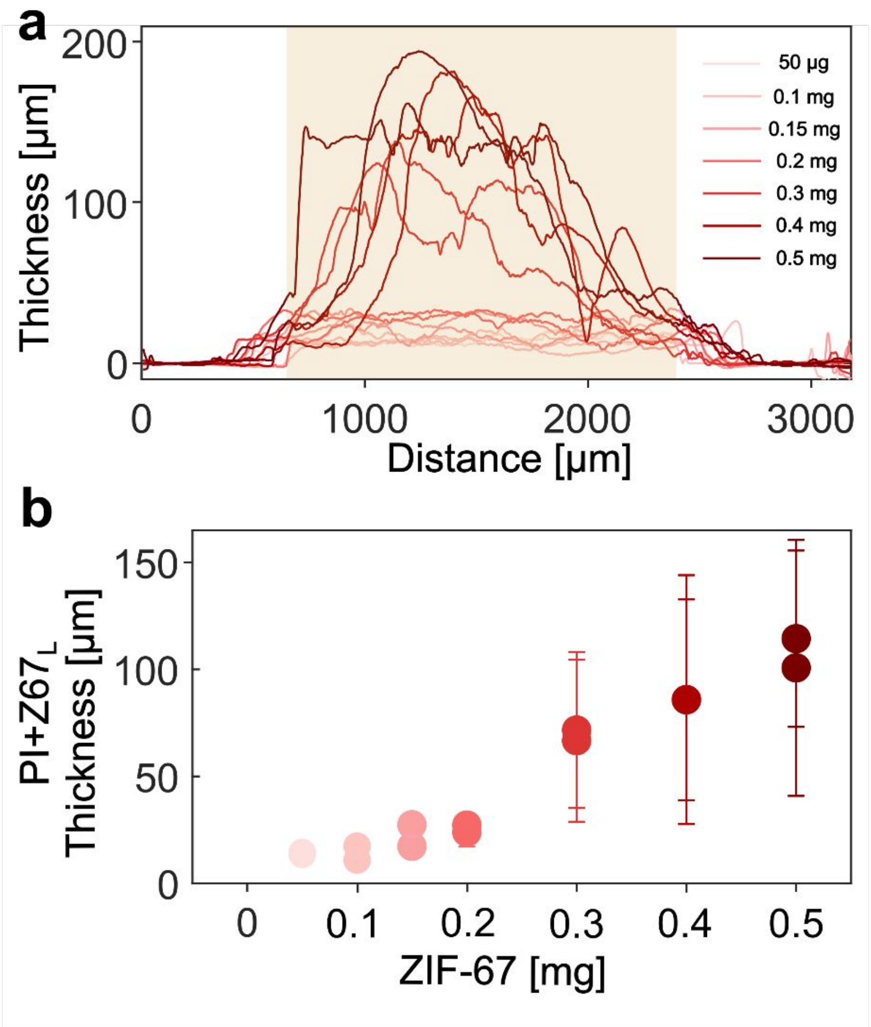

Figure S17. ZIF-67 loading. (a) Thickness profiles of PI+Z67$_L$ electrodes with varying amounts of ZIF-67 (ranging from 50 µg to 0.5 mg) deposited on the sensing area. As can be seen from the profile in the top image, the ZIF-67 coating is uniform from 50 µg to 0.2mg (4x 50µg), while ZIF-67 loadings exceeding 0.2 mg lead to non-uniform thickness distribution and compromised mechanical stability of the coating. (b) PI+Z67 average thickness vs. ZIF-67 loading. The electrode thickness increases proportionally with the amount of ZIF-67 loaded, as evidenced by

the profile measurements, becoming unstable beyond 0.2 mg of ZIF-67 as shown by the increase of the standard deviation.

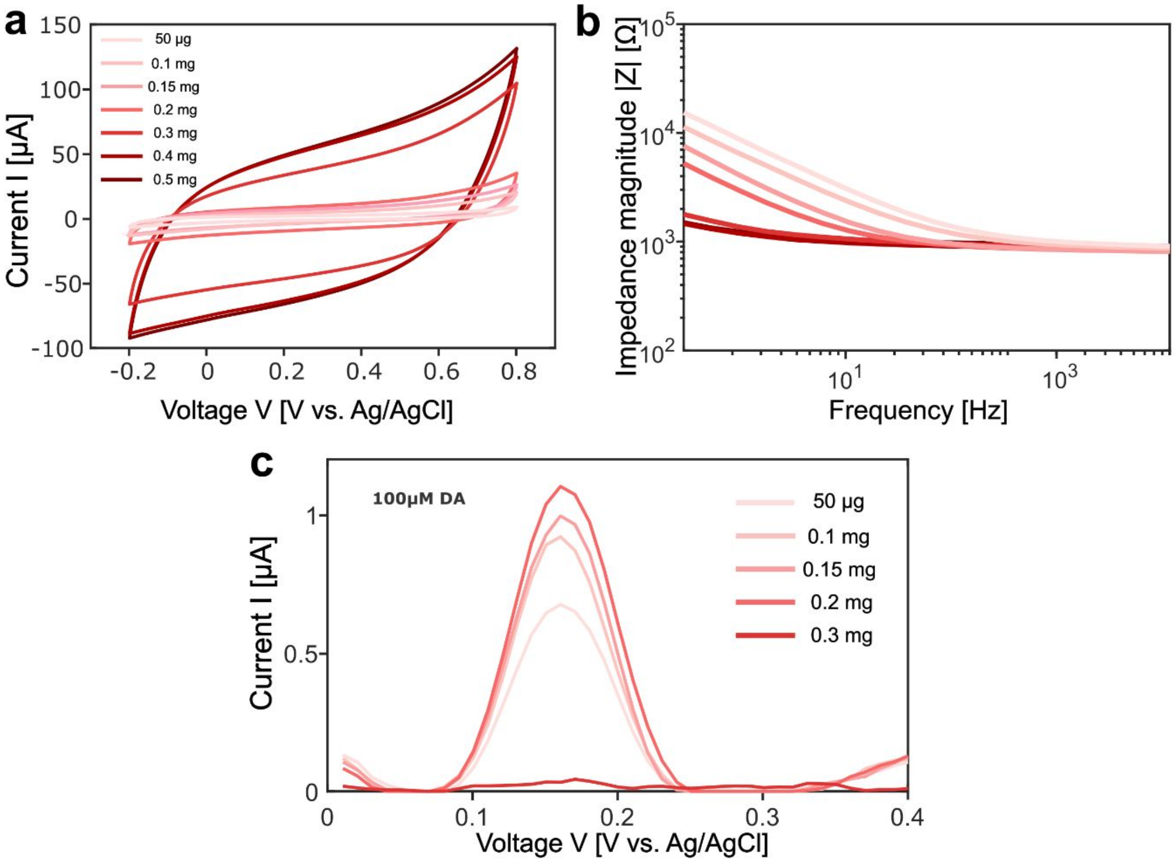

Figure S18. (a) Cyclic voltammograms recorded in a PBS solution from PI+ZIF-67_L with different ZIF-67 loading (50mg, 1x, 2x, 3x, 4x, 6x, 8x, 10x). The third cycle is plotted for the CVs. As shown, higher ZIF-67 loadings correspond to increased current responses. (b) The corresponding impedance spectroscopy is shown. (c) DPV response for dopamine (100 µᴍ) using different loading of ZIF-67 (50mg, 1x, 2x, 3x, 4x, 6x). As shown, the sensitivity to dopamine increases with higher ZIF-67 loadings up to 0.2 mg. At 0.3 mg of ZIF-67, dopamine is no longer detected. The loss of sensitivity at higher loadings is attributed to higher capacitance, which may interfere with

the faradaic response detection, and the increased electrode thickness that results in longer diffusion times for dopamine. Based on these results, 0.2 mg of ZIF-67 is selected as the optimal loading for sensor fabrication.

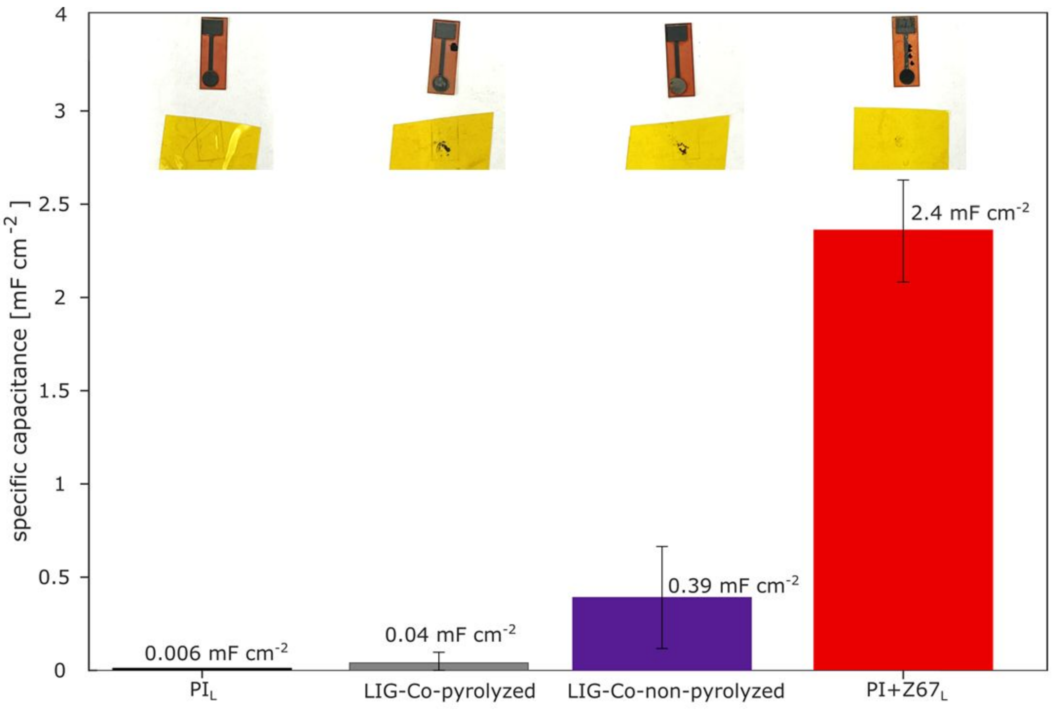

Figure S19. To compare the performance of ZIF-67 derived $Co/Co_3O_4/C$ with commercially available cobalt nanoparticles (NP_S), identical electrodes were prepared either laser-pyrolyzed or non-pyrolyzed (as-deposited). An identical method of ink preparation and catalyst loading was followed to prepare commercial Co NPs. In addition, a simple adhesion test (scotch tape method) was performed, and it can be concluded that the $PI+Z67_L$ is the most structurally stable.

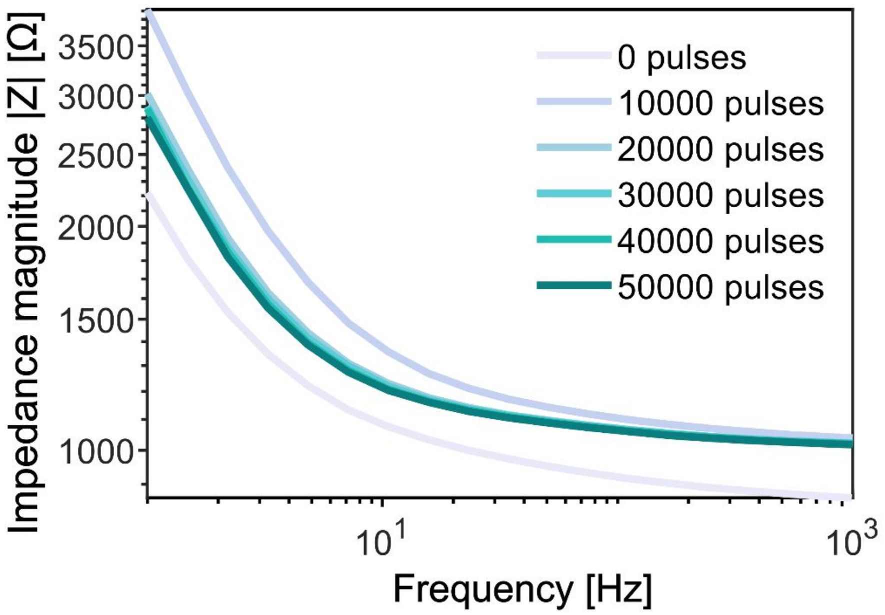

Figure S20. Electrochemical stability of the PI+Z67$_L$, evaluated before (0 pulses) and after 50000 pulses. In this sample, 0.2 mg of ZIF-67 ink was drop-casted on PI substrate before laser scribing.

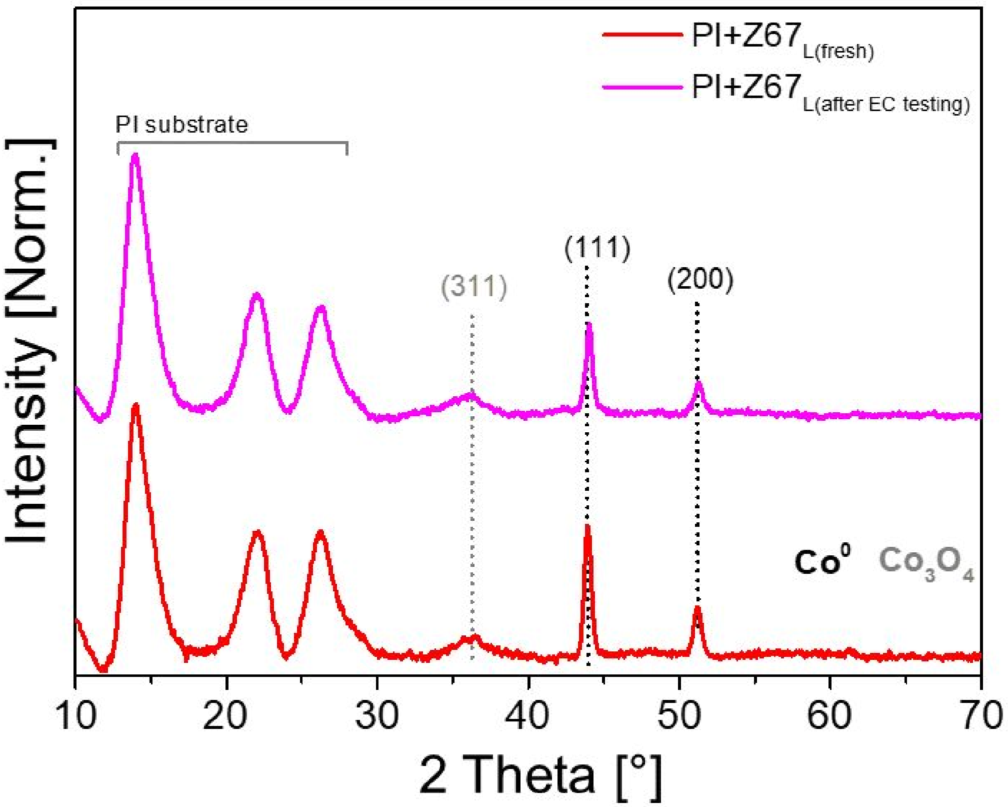

Figure S21. XRD spectra of fresh PI+Z67$_L$ and after using for electrochemical (EC) sensing of dopamine in PBS solution. The spectra show that the graphitic carbon as well as Co/Co_3O_4 exhibit no trivial change after a long exposure to the harsh EC conditions (in PBS solution) which confirms their structural stability.

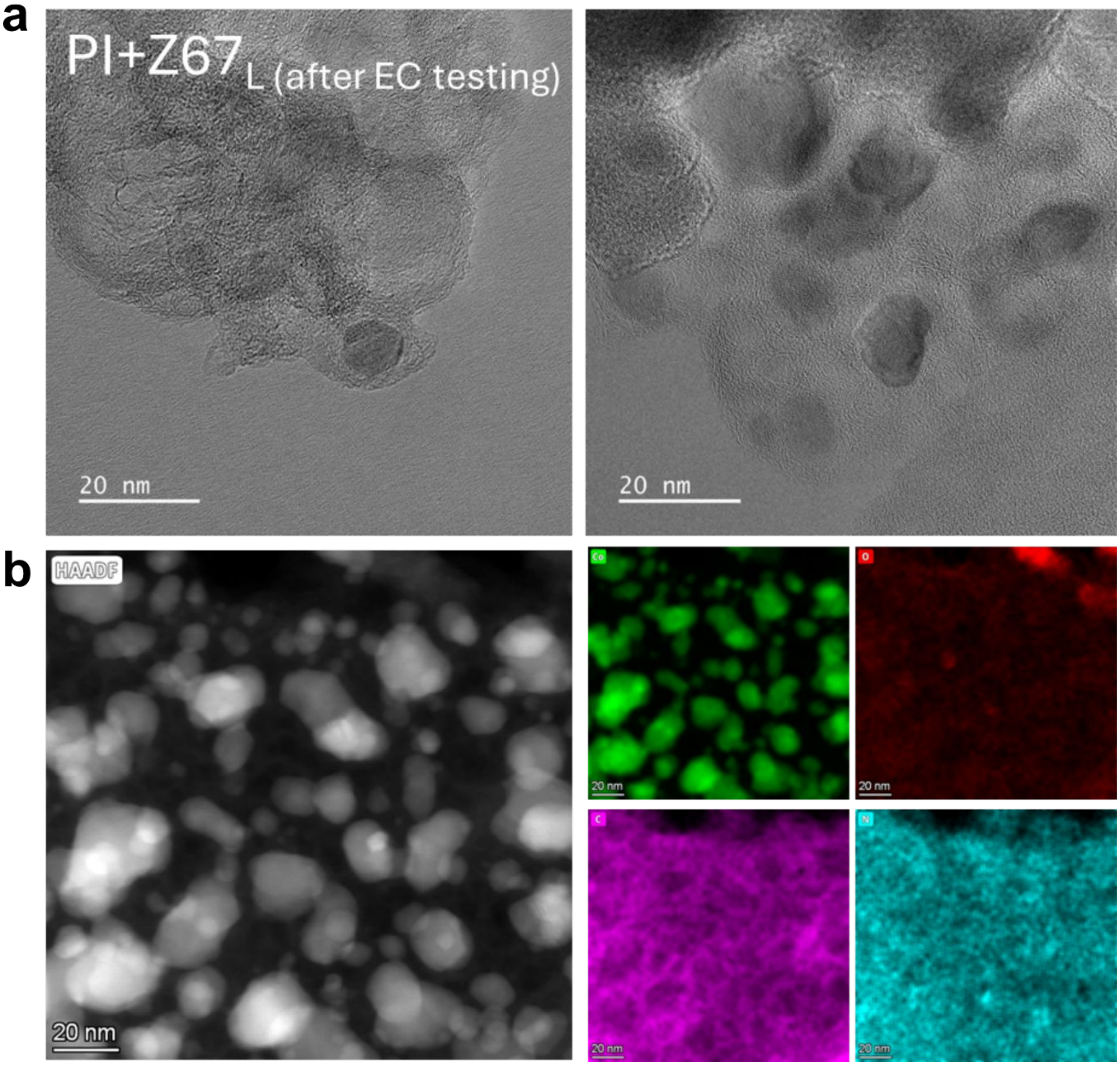

Figure S22. (a) HR-TEM images of Co NPs in carbon matrix after EC testing and (b) HAADF-STEM image showing particle size distribution of Co NPs (left) and STEM-EDX elemental maps of PI+Z67$_L$ after being used for electrochemical sensing of dopamine in PBS. The HR-TEM and STEM-EDX maps of Co, C, O and N confirm the stability of Co/Co_3O_4 NPs in porous graphitic carbon matrix.

REFERENCES

(1) Park, H.; Amaranatha Reddy, D.; Kim, Y.; Ma, R.; Choi, J.; Kim, T. K.; Lee, K.-S. Zeolitic Imidazolate Framework-67 (ZIF-67) Rhombic Dodecahedrons as Full-Spectrum Light Harvesting Photocatalyst for Environmental Remediation. *Solid State Sciences* **2016**, *62*, 82–89. https://doi.org/10.1016/j.solidstatesciences.2016.10.018.

(2) Fettkenhauer, C.; Wang, X.; Kailasam, K.; Antonietti, M.; Dontsova, D. Synthesis of Efficient Photocatalysts for Water Oxidation and Dye Degradation Reactions Using CoCl 2 Eutectics. *Journal of Materials Chemistry A* **2015**, *3* (42), 21227–21232. https://doi.org/10.1039/C5TA06304C.
